# Functionalized Magnetite Nanoparticles: Characterization, Bioeffects, and Role of Reactive Oxygen Species in Unicellular and Enzymatic Systems

**DOI:** 10.3390/ijms24021133

**Published:** 2023-01-06

**Authors:** Arina G. Kicheeva, Ekaterina S. Sushko, Lyubov S. Bondarenko, Kamila A. Kydralieva, Denis A. Pankratov, Nataliya S. Tropskaya, Artur A. Dzeranov, Gulzhian I. Dzhardimalieva, Mauro Zarrelli, Nadezhda S. Kudryasheva

**Affiliations:** 1 Institute of Biophysics of Siberian Branch of Russian Academy of Sciences, Federal Research Center “Krasnoyarsk Science Center” of Siberian Branch of Russian Academy of Sciences, 660036 Krasnoyarsk, Russia; 2 Institute of Physics of Siberian Branch of Russian Academy of Sciences, Federal Research Center “Krasnoyarsk Science Center” of Siberian Branch of Russian Academy of Sciences, 660036 Krasnoyarsk, Russia; 3 Department of General Engineering, Moscow Aviation Institute (National Research University), 125993 Moscow, Russia; 4 Department of Chemistry, Lomonosov Moscow State University, 119991 Moscow, Russia; 5 Sklifosovsky Research Institute for Emergency Medicine, 129010 Moscow, Russia; 6 Federal Research Center of Problems of Chemical Physics and Medicinal Chemistry, Russian Academy of Sciences, 142432 Chernogolovka, Russia; 7 Institute for Polymers, Composites and Biomaterials, National Research Council of Italy, P.le Fermi, 1, 80055 Portici, Italy; 8 Biophysics Department, Siberian Federal University, 660041 Krasnoyarsk, Russia

**Keywords:** magnetite nanoparticles, surface modification, humic acids, organosilane, reactive oxygen species, toxicity, bioluminescence assay, bacteria, enzymes, oxidative stress, prooxidant, ferroptosis

## Abstract

The current study evaluates the role of reactive oxygen species (ROS) in bioeffects of magnetite nanoparticles (MNPs), such as bare (Fe_3_O_4_), humic acids (Fe_3_O_4_-HA), and 3-aminopropyltriethoxysilane (Fe_3_O_4_-APTES) modified MNPs. Mössbauer spectroscopy was used to identify the local surrounding for Fe atom/ions and the depth of modification for MNPs. It was found that the Fe_3_O_4_-HA MNPs contain the smallest, whereas the Fe_3_O_4_-APTES MNPs contain the largest amount of Fe^2+^ ions. Bioluminescent cellular and enzymatic assays were applied to monitor the toxicity and anti-(pro-)oxidant activity of MNPs. The contents of ROS were determined by a chemiluminescence luminol assay evaluating the correlations with toxicity/anti-(pro-)oxidant coefficients. Toxic effects of modified MNPs were found at higher concentrations (>10^−2^ g/L); they were related to ROS storage in bacterial suspensions. MNPs stimulated ROS production by the bacteria in a wide concentration range (10^−15^–1 g/L). Under the conditions of model oxidative stress and higher concentrations of MNPs (>10^−4^ g/L), the bacterial bioassay revealed prooxidant activity of all three MNP types, with corresponding decay of ROS content. Bioluminescence enzymatic assay did not show any sensitivity to MNPs, with negligible change in ROS content. The results clearly indicate that cell-membrane processes are responsible for the bioeffects and bacterial ROS generation, confirming the ferroptosis phenomenon based on iron-initiated cell-membrane lipid peroxidation.

## 1. Introduction

Magnetic nanoparticles (MNPs) are of wide scientific interest due to their potential applications in biology, medicine, nanotechnology, and the environment. These applications include enzyme and protein separation, RNA and DNA purification [1,2,3], controlled and targeted drug delivery [4,5], immunoassay [6], biosensor production for toxic pesticide detection [7,8], a contrast agent for magnetic resonance imaging [9,10], hyperthermic therapy for cancer diagnosis [11,12], and cancer treatment [13,14]. Another important application is the removal of ecotoxicants from natural waters and industrial wastes [1,15]. Therefore, iron oxide nanomaterials can be found in natural and engineering aquatic environments due to their numerous applications, as well as corrosion products of metal components in the presence of natural materials [16,17].

With the development of nanotechnology, iron-based nanoparticles have become widely used in cancer therapy research. A new, iron-dependent form of programmed cell death, ferroptosis, first described in 2012 by Dixon [18], is one of the types of regulated cell death caused by strong activation of lipid peroxidation, which is concerned with the production of reactive oxygen species (ROS) and iron availability in cells [19].

Ferroptosis-inducing systems initiate the overexpression of H_2_O_2_ in a cancer cell [20,21] and the subsequent formation of hydroxyl radicals. Among the ferroptosis-inducing systems, the following substances are known: complexes [22,23,24], various iron (II, III)-based nanoplatforms [25,26,27], including ferromagnetic nanoparticles (maghemite γ-Fe_2_O_3_ or magnetite Fe_3_O_4_) [28], iron-containing nanometallic silicas [29] and metal-organic frameworks [30]. The study of the release of iron ions is associated with the possibility of newly formed iron ions affecting the intracellular redox reactions and homeostasis of ROS inside cells considerably [31].

In general, ROS are active intermediate compounds involved in the processes of aqueous solutions. ROS are natural by-products of cellular oxidative metabolism; they play an essential role in cell death, modulation of cell survival, differentiation, and cell signaling [32,33]. ROS result from the energy/electron transfer to oxygen; they are highly reactive and potentially harmful to living organisms [34]. The group of ROS includes free radicals such as singlet oxygen, superoxide, hydroxyl radical, etc., as well as radical precursors, such as hydrogen peroxide H_2_O_2_, a compound of longer lifespan and higher stability as compared with free radicals [35,36]. ROS are important intermediates in certain physiological processes (e.g., respiration, cell signaling), and their cell levels are rigorously controlled via antioxidants of different types—enzymatic (e.g., catalase, glutathione transferases, glutathione peroxidase, and superoxide dismutase) and non-enzymatic (e.g., ascorbic acid, tocopherols, cysteine, glutathione, carotenoids, bilirubin, flavonoids). However, this redox homeostasis can be disturbed in some conditions, and overage of ROS may cause oxidative stress, which induces harmful effects to cells through oxidative damage of biomolecules (e.g., proteins, lipids, and nucleic acids) or disruption in cell signaling mechanisms [37]. Iron is inextricably linked to ROS due to its partially filled d-orbitals, variable oxidation states, and involvement in energy transfer or electron-transfer processes. Thus, studying the ROS-related bioactivity of metal nanoparticles defines their application as engineered nanostructures, as well as explains their biological and ecological role as nanostructures of natural origination [38,39].

Pristine MNPs require a protective coating since they can be easily oxidized in air and aggregated after production, especially in aqueous systems [40,41]. Thus, stabilization of iron oxide particles is desirable, it is achieved by functionalizing the MNP surfaces with various substances—polymers, metals, inorganic, and organic substances [42]. In the current paper, we use three types of MNPs: bare Fe_3_O_4_ (1), functionalized with humic acids (2), and alkoxysilane (3).

Humic acids (HA), natural high-molecular organic multifunctional substances, with the domination of carboxyl and hydroxyl groups, are relatively small molecules held together by weak non-covalent intermolecular interactions (e.g., π-π, van der Waals, charge transfer, and hydrogen bonds) among organic fragments [27]. HA are of high affinity to magnetite (Fe_3_O_4_) and effectively cover pristine MNPs, most likely through surface complexing ligand exchange reactions [43,44,45]. HA-modified magnetite can have anti- and pro-oxidant features due to the presence of donor-acceptor groups of HA and magnetically controlled properties due to Fe_3_O_4_ cores [46,47,48]. HA sorption onto Fe_3_O_4_ nanoparticles increases the stability of nanodispersions, preventing their aggregation [49] and potentially increasing the adsorption capacity and selectivity of the nanoparticles [42,50]. The HA-coated MNPs were previously used to remove oxyanions (chromates) of metals, anionic phosphate [42,51], metal cations [52], rhodamine [53], toxic inorganic forms of arsenic [44], for adsorption and reduction of toxic Cr(VI) to non-toxic Cr(III) [51], adsorption of Pb(II), Cu(II), Cd(II), and Ni ions from wastewater [54], and protein immobilization [55].

The possibility to attain functionalized iron-based nanoparticles by alkoxysilanes is of wide scientific interest to researchers [56]. The chemistry of the silica surface is well established, enabling a large number of applications of silica-coated nanoparticles [56,57]. Alkoxysilane can bind to metal oxide by adsorption or covalent linking. A functional alkoxysilane consists of at least two functional groups, the first group being used for attachment to the surface of the MNPs, and the second group providing an adhesion point to the bond of the molecule [1]. Nanoparticles coated with silica or alkoxysilanes are promising reagents in nanobiotechnology due to their biocompatibility and hydrophilic properties. The great variety of alkoxysilanes makes it possible to carry out various types of functionalization of the surface of nanoparticles by introducing charges into the system. Thus, functionalization prevents from the aggregation of nanoparticles in liquids and improves their chemical stability by controlling surface charge [58].

In this study, we chose a luminous marine bacterium as a model microorganism to evaluate and compare the biological effects of MNPs. The bioluminescence bacteria-based assessment is known as a toxicity assay and it has been applied for more than 50 years for toxicity monitoring due to its simplicity and high sensitivity [59,60,61,62,63,64]. The bioassay uses luminescence intensity as a physiological test parameter providing a convenient approach with high rates of analysis (1–10 min), low costs, and ease of the bioassay procedure.

We compare the cellular bioeffects of MNPs in the bioluminescence enzymatic assay system, proposing a relatively new approach in a toxicology practice [65,66,67,68]. The conventional enzymatic bioluminescent assay is based on the bacterial bioluminescent enzyme system, which involves two coupled enzymatic reactions:

(R1) $$NADH+FMN\overset{NADH:FMN-oxidoreductase}{\to }FMN\cdot H^{-}+NAD^{+}$$

(R2) $$FMN\cdot H^{-}+RCHO+O_{2}\overset{luciferase}{\to }FMN+RCOO^{-}+H_{2}O+h\nu_{\;}$$

where, *FMN* is flavin mononucleotide, *NADH* is nicotinamide adenine dinucleotide disodium salt-reduced.

Similar to cellular bioassay, the enzymatic analysis can assess the general toxicity in test samples allowing the integration of all the interactions of toxic compounds with the bioluminescent system such as redox processes, polar and nonpolar binding, etc. Moreover, the enzymatic bioassay is specific to oxidizers due to its additional kinetic parameter, the induction period, which directly depends on the redox potentials of toxicants in solutions [67]. Previously [69,70], we used the bioluminescent enzymatic assay system to evaluate the toxicities of general and oxidative types in solutions of organic and inorganic oxidizers (quinones and polyvalent metals, respectively). Changes in general toxicity and oxidative toxicity of aqueous media under exposure to humic substances were studied previously in our previous studies [71,72,73]. Later [74,75,76,77,78,79], the toxicity and antioxidant activity of a series of fullerenols (i.e., carbon nanostructures, water-soluble derivatives of fullerenes, and perspective pharmaceutical agents) were evaluated and compared.

In our previous work [80], we used bioluminescence bioassays, cellular and enzymatic, to evaluate the bioeffects of three types of MNPs: bare MNPs, modified by humic acids, and silica (3-aminopropyltriethoxysilane, APTES), i.e., (1) Fe_3_O_4_, (2) Fe_3_O_4_/HA, and (3) Fe_3_O_4_/APTES, respectively. The bioeffects under study were: toxic effects and anti/pro-oxidant activity of the MNPs. Current work continues the study of three types of MNPs [80]; it evaluates the role of ROS in the bioeffects of these nanoparticles. Contents of reactive oxygen species (ROS) were determined using chemiluminescence luminol assay in the bioluminescence assay systems. Correlations of ROS content with toxicity/anti-(pro-)oxidant coefficients were evaluated, thus contributing to comprehending ferroptosis phenomenon.

## 2. Results and Discussion

### 2.1. Characterization of the Microstructure and Magnetic Properties of Samples

#### 2.1.1. Local Environment of Fe Atom/Ions

The composition and morphological properties of MNPs were studied by Mössbauer spectroscopy, and related spectra, along with their description and results of simulation in different state superparamagnetic relaxation models are presented in Table 1 and Supplementary materials (Figure S1, Table S1). Relying on the presented results, it can be argued that magnitudes of magnetic splittings, obtained even for spectra at low temperatures, are lower than expected for massive samples of magnetite and maghemite [81], which is typical for nanosized materials [82]. Indeed, the sizes of magnetic domains can be estimated in the range from 12.6 (for Fe_3_O_4_-HA) to 15.7 (for Fe_3_O_4_-APTES) nm as reported in Table 1.

Moreover, by analyzing the areas of the subspectra related to iron atoms in different crystallographic sites of magnetite, the value of the non-stoichiometric parameter–δ for nano-magnetite–Fe_3−δ_O_4_ ≡ (Fe^3+^)_A_(Fe^2+^_1−3δ_Fe^3+^_1+2δ_#_δ_)_B_O_4_ [15,83], can be estimated by using the following expression:

δ = {Σ(δ_2_ − 3δ_i_ + 2δ_3_)∙S_i_ + (δ_2_ − δ_3_)ΣS_j_}/{Σ(3δ_2_ − δ_i_ − 2δ_3_)∙S_i_ + 3(δ_2_ − δ_3_)ΣS_j_}, (1)

where S_i_ is the relative area of the subspectrum with isomeric shift δ_i_ related to the iron atoms in the B-site, S_j_ is the relative area of the remaining subspectra, δ_2_ and δ_3_ are the isomeric shifts of iron atoms (+2) and (+3) in the octahedral oxygen environment for given temperature (here δ_2_ = 1.16 ± 0.06 и 1.33 ± 0.09 mm/s for 296 and 78 K, respectively, δ_3_ = 0.37 ± 0.04 and 0.49 ± 0.04 mm/s for 296 and 78 K, respectively) [84]. Computed results are reported in Table 1, and the data reveal that magnetite samples are quite strongly oxidized indicating a magnetite-maghemite solid solution [15]. At the same time, the Fe_3_O_4_-HA sample contains the smallest amount of Fe^2+^ ions, whereas the Fe_3_O_4_-APTES contain the largest amount. It should be noted that, in the case of the studied samples, there is a clear correlation between their particle sizes and nonstoichiometric composition which leads to the following result: the smaller the particle size, the more nonstoichiometric the nanomagnetite composition is (Table 1).

#### 2.1.2. Magnetic Parameters

**Figure 1:**
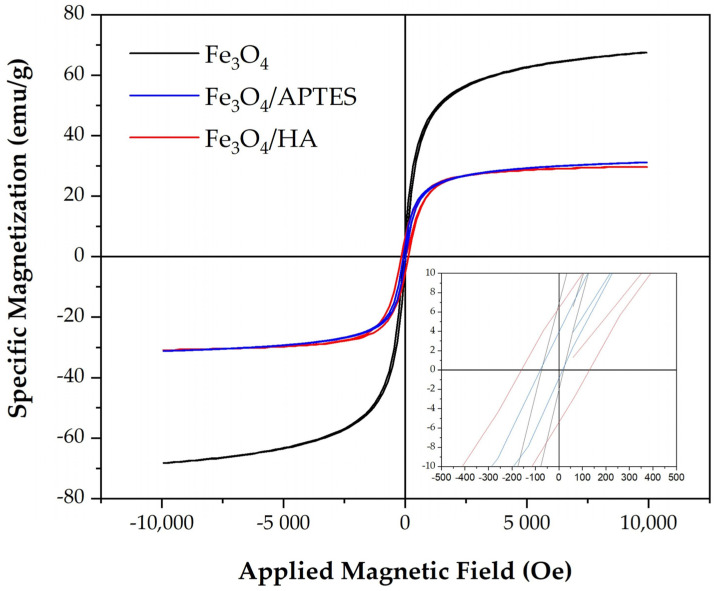
Hysteresis loops at 300 K for Fe_3_O_4_, Fe_3_O_4_/HA, and Fe_3_O_4_/APTES. The inset shows an enlarged view of the data at lower magnetic fields. The surface modification led to a twofold decrease in the saturation magnetization.

The most crucial property of MNPs, which allow a variety of applications, especially in biomedicine, is represented by their ferrimagnetism. As a matter of fact, the application of an external magnetic field makes it possible to concentrate magnetic nanoparticles in the target point, thus reducing likely side effects [85,86,87,88]. Some magnetic characteristics for the Fe_3_O_4_ MNPs are presented in Table 1. The hysteresis loops for the three different magnetite NMPs results were closed and symmetrical with respect to the origin of the coordinate system as reported in Figure 1.

The shape of the loops indicates the ferromagnetic features of the material which are prone to their potential application. The saturation magnetizations of bare Fe_3_O_4_, Fe_3_O_4_/HA, and Fe_3_O_4_/APTES were respectively 68.2, 30.9, and 31.2 emu/g (Table 1), suggesting a 40% (*w*/*w*) of HA content in Fe_3_O_4_/HA. The attained values of saturation magnetization indicate that NMPs are stabilized by the HA or APTES functionalization exhibiting superparamagnetic properties at room temperature (Figure 1) and the corresponding variations, respectively of 54.6% for the Fe_3_O_4_/H and 54.5% for the Fe_3_O_4_/APTES, compared to bare magnetite can be explained by the noncollinearity of surface spins of MNPs and likely by the same thickness of the HA and APTES shell surrounding the nanomagnetite particles. In the absence of a magnetic field, all samples showed a similar low residual magnetism ~±4–7 emu g^−1^ (Table 1), due to magnetic viscosity for superparamagnetic materials [89]. The further increase of the coercivity can be related to cumulative anisotropy by phase transformation to maghemite in the case of Fe_3_O_4_/HA in terms of concentration compared to Fe_3_O_4_/APTES, which correlates with Mössbauer spectroscopy data [90].

In general, bare and functionalized nanoparticles exhibit a saturation magnetization sufficient to control an external magnetic field. In many works, focused on the preparation of materials for catalyzing ferroptosis, the authors have used iron salts as iron ion sources [29,91,92], however, as reported by Huo et al., 2017, [93] without targeting, only 6.95% of nanoparticles were stored and localised in the 4T1 cancer cell 48 h upon injection.

#### 2.1.3. Iron Ion Release

The study of the release of Fe^2+^ ions and Fe^3+^ ions is also associated with the different contributions of ions of different redox states to the Fenton reaction [94]. It is known that the rate constant of •OH generation via Fenton reaction catalyzed by Fe^2+^ ion [76 (mol/L)^−i^ s ^−s^] is 10^4^ −10^5^ times higher than this of the Fe^3+^ ion-catalyzed Fenton-like reaction [0.001−0.07 (mol/L)^−i^ s ^−s^] [94]. In this regard, estimation of the concentration of released ions of different redox states is of interest in order to identify their contribution to the induction of ROS production.

**Figure 2:**
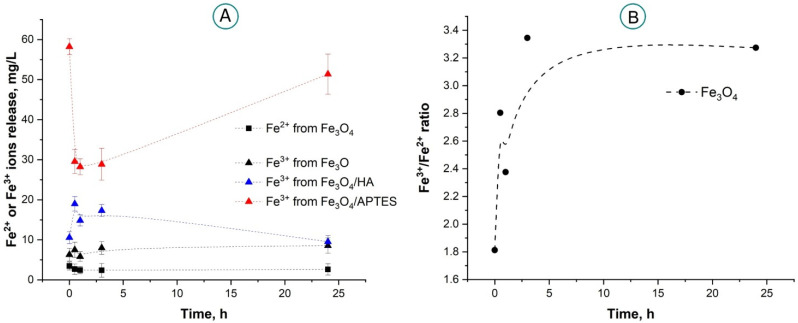
Kinetics of release of iron ions by MNPs (**A**,**B**) (mg/L of ions from 1 g/L of Fe in the sample). Colors denote various nanoparticles, “square” markers denote the release of Fe^2+^ ions, and “triangle” markers denote Fe^3+^ ions. According to the obtained data, samples of Fe_3_O_4_/HA and Fe_3_O_4_/APTES release only Fe^3+^ ions, while Fe_3_O_4_ releases both Fe^2+^ and Fe^3+^ ions (**A**), and their ratio increases within three hours reaching a plateau (**B**).

UV-Vis spectroscopy was used to investigate the kinetics of the release of Fe^2+^ and Fe^3+^ ions from nanoparticles Fe_3_O_4_, Fe_3_O_4_/HA, and Fe_3_O_4_/APTES (Figure S2). It is noticeable from the spectra, that Fe_3_O_4_ suspension reveals a release of both Fe^2+^ and Fe^3+^ ions, respectively of 6.3 mg/L and 3.5 mg/L, as shown in Figure 2A. The Fe^3+^/Fe^2+^ ratio during the first hour was close to the stoichiometric ratio for magnetite, i.e., 1.8–2.3 (Figure 2B). Upon 24 h, Fe^3+^ concentration increases taking the Fe^3+^/Fe^2+^ ratio to a value of 3.3 due to the oxidation of Fe^2+^ to Fe^3+^ ions.

Fe_3_O_4_/HA and Fe_3_O_4_/APTES do not release Fe^2+^ ions within 24 h, Figure 2A. In the case of Fe_3_O_4_/HA, the absence of Fe^2+^ ions within the solution can be explained by the complete oxidation of magnetite Fe_3_O_4_ to maghemite Fe_2_O_3_, according to the Mössbauer spectroscopy data (Section 2.1.1). However, analyzing the same data as well as results of X-ray diffraction [82], APTES-functionalized nanoparticles contain both Fe^2+^ and Fe^3+^ ions. The absence of Fe^2+^ ions into the Fe_3_O_4_/APTES solution can be explained by the very low concentration which can fall beyond the sensitivity limits of the employed analysis technique. Additionally, Fe^2+^ would undergo spontaneous chemical oxidation with molecular oxygen or other potential oxidants to Fe^3+^, which eventually precipitate as ferric hydroxides [95,96].

Both functionalized oxides, Fe_3_O_4_/HA and Fe_3_O_4_/APTES, release Fe^3+^ ions, however, the release kinetics differ significantly (Figure 2A). So, in the case of Fe_3_O_4_/HA, the Fe^3+^ concentration in the solution increases from 10.6 mg/L to 19.0 mg/L in a half an hour, and then decreases to 9.5 mg/L in 24 h. In the case of Fe_3_O_4_/APTES, on the contrary, a decay of Fe^3+^ release (from 58.2 mg/L to 29.6 mg/L in half an hour after suspension) was revealed with a subsequent increase of Fe^3+^ concentration almost to the initial value of 51.4 mg/L after 24 h. In general, functionalized MNPs release more Fe^3+^ ions compared to bare MNPs, but do not or negligibly release Fe^2+^ ions. For this reason, bare MNPs represent an effective source of a pair of Fe^2+^/Fe^3+^ ions, whereas the HA and APTES functionalized nanoparticles, under the specified synthesis conditions, are a source of only the Fe^3+^ ions.

The difference in the behavior of iron ions released from the different considered samples would need an attempted explanation. In the case of bare Fe_3_O_4_, their occurring aggregation leads to a decrease in the concentration of surface ions and consequently to their hydrolysis forming stable hydrocomplexes as supported by the binding constant increasing for lgK(Fe(OH)_3aq_) and lgK(Fe(OH)_4_), from 12 and 22, respectively [97]. Due to a high complexing potential of HA, the binding constants (lgK) of Fe^2+^ and Fe^3+^ ions are, five and four, respectively, according to [98]. Based on this evidence, HA functionalization not only induces stabilization of the magnetite, but also triggers iron ions to be pulled out by HA from the NP’s surface leading into the solution. The higher release of iron ions, in the case of Fe_3_O_4_/APTES, is probably associated with a high level of porosity and amorphous structure of silica, according to the results reported by Oliveira, et al., 2019, and Otalvaro, et al., 2019, [99,100].

In 1987, Minotti et al. [101] showed that lipid peroxidation can be induced both by the participation of Fe^2+^ or Fe^3+^ ions in the formation of hydroxyl radicals •OH as a result of the Fenton reaction, and also by the participation of pairs of Fe^2+^/Fe^3+^ ions immediately to form the Fe^3+^-dioxygen-Fe^2+^ complex. However, in a physiologically neutral or slightly acidic environment such as a tumor area, the effectiveness of the Fenton reaction is relatively low [102]. Even under acidic pH conditions, the Fenton reaction catalyzed by Fe^2+^ has a low reaction rate (~63 M^−1^ s^−1^), leading to the slow formation of ROS [103]. For these reasons, the development of nanopreparations featured by high catalytic activity and specificity, is highly prospective in a weakly acidic and neutral media (pH 6–7) as a tumor microenvironment.

The ability to generate ROS by MNPs in highly diluted solutions was studied in relation to bacterial and enzymatic systems at a pH value of ~6 in Section 2.2 and Section 2.3.

### 2.2. Effects of MNPs on Bacterial Cells

The luminescence intensity of bacterial cells was studied in the presence of MNPs considering different concentrations of MNPs. The MNP effects were analyzed at <1 g/L, as higher content was limited by both low water solubility and nanoparticles dispersion stability during the experiments and also by the demand to avoid the “optical filter” effect, which limits the use of luminescence signal detection in solutions with high optical density and/or light-scattering suspensions [104].

#### 2.2.1. Toxic Effects of MNPs in Bacterial Suspension

(a) Effects of MNPs on Bacterial Bioluminescence Intensity

**Figure 3:**
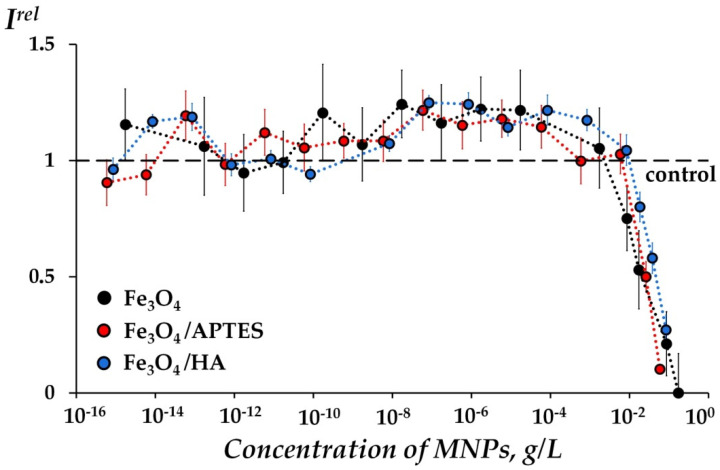
Relative bioluminescence intensity, *I^re^*^l^, in bacterial suspension vs. MNP concentration, g/L. Bare Fe_3_O_4_–black circles, Fe_3_O_4_/APTES–red circles, and Fe_3_O_4_/HA–blue circles.

All three types of MNPs demonstrated moderate bioluminescence activation (1.0 *< I^rel^ <* 1.25, *p* < 0.05) in a lower concentration range (i.e., 10^−8^–10^−2^ g/L), as shown in Figure 3. The activation of bacterial response by MNPs might be described in terms of the conventional “hormesis” model [105,106,107,108]. It is known that the model includes, in the broadest case, three stages of the biological dose-dependent response—stress recognition (I), activation (II), and inhibition of organismal functions or toxic effect (III). As a concept, hormesis involves favorable biological responses to low exposures to stressors, in all cases [109,110].

To characterize the toxicity of the MNP samples, the bioluminescence inhibition (*I^rel^* < 1.0, *p* < 0.05) effects due to higher concentrations of MNPs (>10^−2^ g/L) were analyzed. The *EC*_50_ values of MNPs (i.e., MNP concentrations at 50% bioluminescence inhibition) were determined and presented in Table 2. The results show that the values are comparable, however, the lowest value of *EC*_50_ of bare Fe_3_O_4_ is evidence of its higher toxicity compared to surface-modified MNPs (Fe_3_O_4_/APTES and Fe_3_O_4_/HA). Probably, this is a result of the Fe^2+^ release in suspensions of bare Fe_3_O_4_, the participation of Fe^2+^/Fe^3+^ pairs (see Section 2.1.3, Figure 2A,B), which actively produce toxic hydroxyl radicals (according to Fenton reaction [101]) and the result in lipid peroxidation in the cellular membrane.

(b) Effects of MNPs on ROS Content in Bacterial Suspension

**Figure 4:**
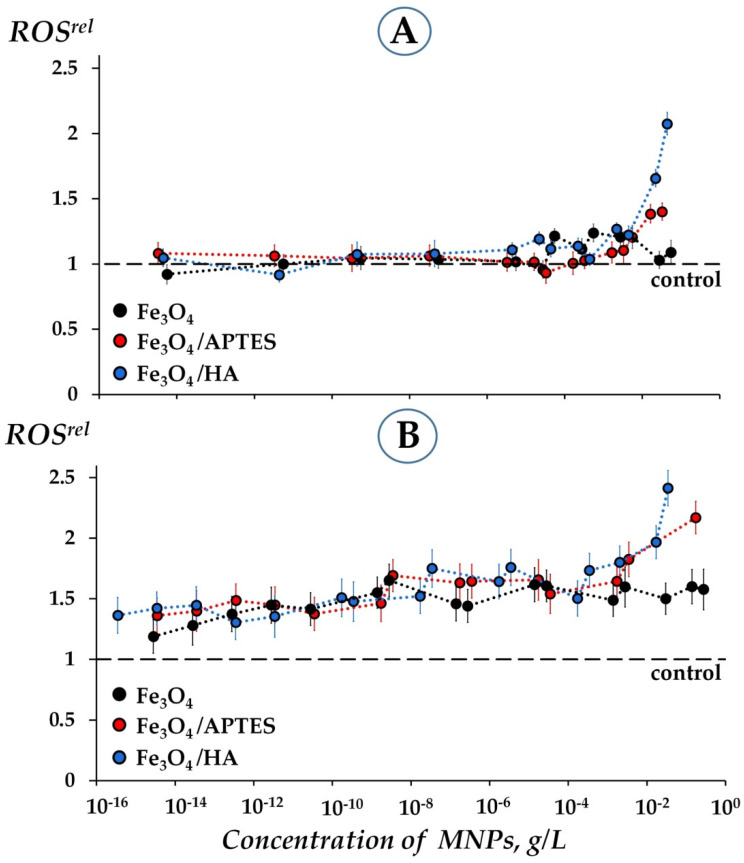
Relative ROS content, *ROS^rel^*, at various concentrations of MNPs in suspensions of two types: (**A**) bacteria-free solutions, (**B**) bacterial suspensions. Contents of ROS in the control samples were ~3 × 10^−6^ M in both bacteria-free (**A**) and bacterial (**B**) media. Bare Fe_3_O_4_–black circles, Fe_3_O_4_ /APTES–red circles, and Fe_3_O_4_/HA–blue circles.

ROS content was determined for two types of suspensions: (1) MNPs in bacteria-free solution (Figure 4A) and (2) MNPs + bacteria (Figure 4B).

Figure 4A shows an ROS increased content in bacteria-free media at higher concentrations of MNPs (10^−4^–10^−1^ g/L) and only a negligible rise was observed for bare Fe_3_O_4_. Whereas, a relevant variation was observed for both modified MNPs at >10^−3^ g/L. Such difference between ROS formation into MNP suspensions between modified and non-modified surface MNPs could be probably related to free Fe^3+^ ions content in the suspension, Figure 2A.

Similar differences in ROS content due to modified and unmodified MNPs were assessed in bacterial suspensions at high nanoparticle concentrations (>10^−3^ g/L, refer to Figure 4B). In fact, an increase in the ROS content was found at high concentrations (>10^–3^ g/L) of two modified MNPs, but not observed for bare Fe_3_O_4_. Similarly to the bacteria-free media (Figure 4A), this may be due to a higher concentration of Fe^3+^ released by functionalized nanoparticles (Figure 2A).

It should be noted, that a moderated increase in the ROS content (as compared to the control samples, *ROS^rel^* > 1.0, *p* < 0.05) was observed over a wide range of MNP concentrations (10^–15^–10^–3^ g/L) in bacterial media (Figure 4B) compared to the bacteria-free media (Figure 4A). This clearly indicates the effective ROS generation associated with the bacterium itself within the low-concentration solutions of MNPs. The above-mentioned effect represents an important achievement that should be further studied in detail.

Correlations between bioluminescence intensity *I^rel^* and *ROS^rel^* (Figure 3 and Figure 4B) were analyzed. The correlation coefficients, *r*, were calculated for three MNP types, as reported in Table 3. High reversed correlations (*r* = −0.88; −0.79) for two modified MNPs (Fe_3_O_4_/APTES, Fe_3_O_4_/HA) were demonstrated, whereas a low correlation (*r* = −0.21) was determined for bare Fe_3_O_4_. The high level of correlations is indicative of a high ROS contribution to the bioluminescence-inhibiting (“toxic”) effect of the modified MNPs within the bacterial media. The effect of modified MNPs related to the ROS involvement could be associated with Fenton-like reactions (catalyzed by Fe^3+^ ion) or Haber−Weiss reaction at neutral pH, whereas the low correlation of bare MNPs is a reasonable indication of additional mechanisms of bacterial inhibition. Supposedly, the Fe^2+^ presence and lower Fe^3+^ concentration in the suspensions of bare MNPs (as shown in Section 2.1.3, Figure 2A) lead to the reasonable assumption of additional occurring mechanisms, such as the production of toxic −OH via Fenton reaction [101] and break of quantitative correlations between ROS and MNP contents (Table 3). 

Previously, high positive correlations between *I^rel^* and *ROS^rel^* were found in suspensions of luminous bacteria and fullerenol at high ”toxic” fullerenol’ concentrations [79]. Fullerenols, conversely to MNPs, are antioxidants and active trappers of free radicals, and they could lower the ROS content effectively [111,112,113,114,115,116] suppressing the rates of oxidative processes in cells. Hence, the opposite signs of correlations for fullerenols and MNPs could be concerned with their anti- and pro-oxidant properties, respectively, indicating the complexity and multiplicity of processes of different bioactive compounds.

#### 2.2.2. Effects of MNPs in Bacterial Suspension under Model Oxidative Stress

The MNP anti-(pro-)oxidant activity in bacterial suspensions was investigated under specific conditions of model oxidative stress. During this study, toxic concentrations of MNPs (i.e., >10^−2^ g/L) were excluded, while bacterial bioluminescence kinetics was monitored and recorded assuming model solutions of an oxidizing agent, 1,4-benzoquinone (Bq), at its *EC*_50_ to suppresses bioluminescence intensity by 50% (*EC*_50_, *_Bq_* = 10^–7^ M). The ROS content was monitored by chemiluminescence technique, within the same media after bioluminescence measurements. It has been assumed that restoration of the bioluminescence intensity (*I^rel^ _Bq_* > 1) results in evidence of MNP antioxidant activity, whereas the additional bioluminescence suppression (*I^rel^ _Bq_* < 1) would indicate a prooxidant attitude of MNPs.

(a) Effects of MNPs on Bacterial Bioluminescence Intensity under Model Oxidative Stress

**Figure 5:**
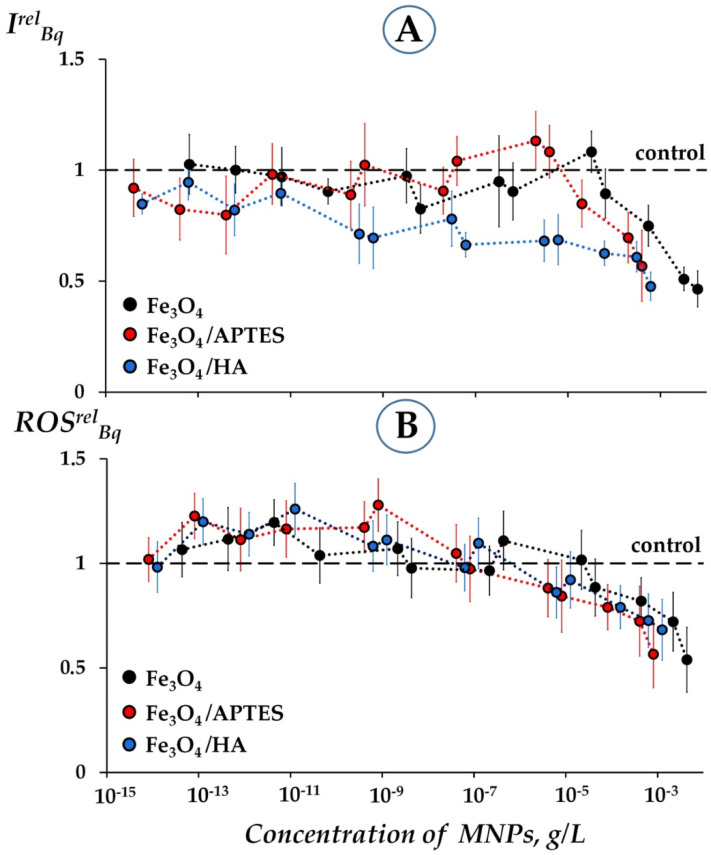
Relative bioluminescence intensity, *I^rel^_Bq_*, (**A**), and relative ROS content, *ROS^rel^ _Bq_*, in the presence of 1,4-benzoquinone (Bq) (**B**) vs. MNP concentration in bacterial suspension under model oxidative stress. The 1,4-benzoquinone concentration was *EC*_50,_*_Bq_* = 10^−7^ M (control) with ROS content of 2.7 × 10^−6^ M. Bare Fe_3_O_4_–black circles, Fe_3_O_4_ /APTES–red circles, and Fe_3_O_4_/HA–blue circles.

Figure 5A shows the *I^rel^ _Bq_*-values at different concentrations of MNPs. All considered MNPs did not activate reliably the bacterial bioluminescence under oxidative stress, hence, it can be reasonably concluded that MNPs do not induce any antioxidant activity. Oppositely, the *I^rel^ _Bq_*-values showed a suppression of the bioluminescence intensity (i.e., *I^rel^ _Bq_* < 1, *p* < 0.05) at higher concentrations of MNPs (>5 × 10^−5^ g/L), confirming the prooxidant properties of MNPs. The HA-modified MNPs (Fe_3_O_4_/HA) suppressed bioluminescence intensity (i.e., *I^rel^ _Bq_* < 1, *p* < 0.05) reliably in a wider range of concentrations (10^–12^–10^–2^ g/L), as compared to Fe_3_O_4_ and Fe_3_O_4_/APTES and this can be explained by the partial hydrolysis of the HA coating in Bq solutions which leads, as a result, to an oxidized iron higher content (Fe^3+^, surface and/or free) in Fe_3_O_4_/HA suspensions.

(b) Effects of MNPs on ROS Content in Bacterial Suspension under Model Oxidative Stress

**Scheme 1:**
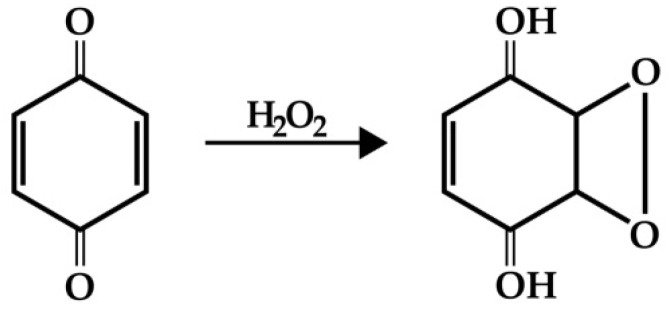
Interaction of 1,4-benzoquinone (Bq) with hydrogen peroxide with peroxide formation.

The ROS content was preliminarily measured in the absence of MNPs at the following different conditions: in physiological saline (3% NaCl) solution, in bacteria-free and in bacterial media under oxidative stress model conditions (i.e., at *EC*_50_ of Bq, *EC*_50,_ *_Bq_* = 10^−7^ M). We showed previously, that Bq lower the ROS content by 10% and by 15% in bacteria-free media and bacterial media, respectively. Analogous results were attained by [79], earlier. The ROS content reduction can be explained considering the peroxides, representative of ROS groups, whose tendency to combine to Bq follows Scheme 1 as presented below.

**Scheme 2:**
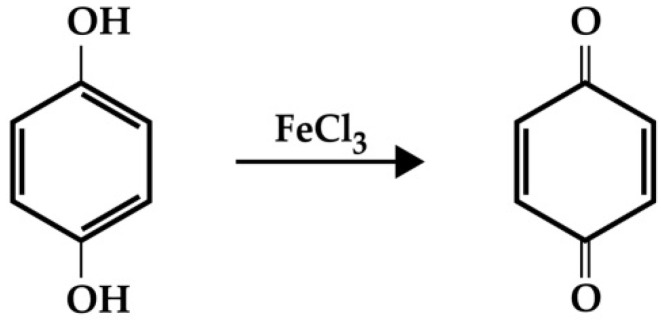
Oxidation of hydroquinone to 1,4-benzoquinone (Bq) with FeCl_3_.

We aimed to reveal how the MNPs addition changes the ROS content in the bacterial suspension under oxidative stress conditions (i.e., in the presence of Bq). Figure 5B shows the ROS content in the bacterial system at various MNP concentrations in the Bq solutions. An additional valuable decay in the content of ROS (*ROS^rel^ _Bq_* < 1, *p* < 0.05) was observed in the concentration range of MNPs 10^−4^–10^−2^ g/L. The further ROS decay might be explained if the Bq balance with its reduced form–hydroquinone–in the bacterial suspension is hypothesized. In this case, the Fe^3+^ addition should shift the balance to the oxidized form, i.e., Bq, according to Scheme 2, and thus decreasing ROS additionally.

Correlation coefficients, *r*, were calculated between the dependencies of *I^rel^ _Bq_* (Figure 5A) and *ROS^rel^ _Bq_* (Figure 5B) for the corresponding MNP concentration range as reported in Table 4. Direct correlations were found, showing direct interrelations between these dependencies. The correlation values confirm interrelations between bioluminescence intensity and ROS under oxidative stress conditions, supporting the envisaged chemical mechanism of free Fe^3+^ involvement (Figure 2A) to the prooxidant activity of MNPs as depicted in Scheme 1 and Scheme 2.

### 2.3. Effects of MNPs on Enzymatic Reactions

The effect of MNPs on bioluminescence enzymatic reaction is a question of interest. Comparison of the MNP effects on bacterial cells and their enzymatic reactions forms understanding at the cellular and biochemical levels, respectively, identifies processes most sensitive to MNP exposure, and allows supposing a role of cellular membrane-related processes in the MNP bioeffects.

Analogously to luminous marine bacteria (Section 2.2.1), the enzyme system luminescence intensity was studied in the presence of Fe_3_O_4_, Fe_3_O_4_/HA, and Fe_3_O_4_/APTES; at low concentrations (i.e., 10^–15^–10^–1^ g/L).

#### 2.3.1. Effects of MNPs without Oxidative Stress

(a) Effects of MNPs on Bioluminescence Intensity of Enzyme Reactions

**Figure 6:**
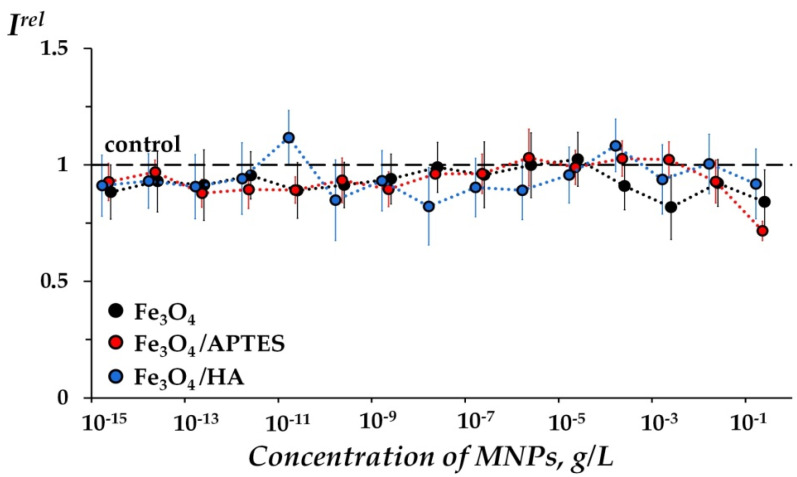
Relative bioluminescence intensity, *I^rel^*, vs. MNP concentration, g/L. Enzyme system. Bare Fe_3_O_4_–black circles, Fe_3_O_4_ /APTES–red circles, and Fe_3_O_4_/HA–blue circles.

Figure 6 presents the dependence of bioluminescence intensity *I^rel^* on MNP concentration in the enzyme system. The plotted data reveal negligible inhibition of the intensity of the bioluminescence.

The difference in MNP effects on the bioluminescence of bacteria and enzymes is evident from the comparison of Figure 3 and Figure 6, respectively. In fact, the MNPs inhibited or moderately activated bacterial bioluminescence, according to Figure 3, but they did not affect the enzymatic bioluminescence as clearly analysing Figure 6. The difference might be related to the complexity of cellular processes, specifically the cell-membrane ones.

(b) Effects of MNPs on ROS Content in Enzyme System

As discussed above for the bacterial system (Section 2.2.1 b), the ROS content was also investigated in the enzyme system using (1) MNPs in an enzyme-free medium and (2) MNPs in enzyme reactions. MNP concentrations were altered as reported in Figure 6, but none of the ROS changes were found for both types of solutions over the entire concentration range (Figures S3 and S4).

#### 2.3.2. Effects of MNPs in Enzyme System under Model Oxidative Stress

To verify the molecular mechanisms of redox MNP effects under oxidative stress conditions in bacteria (Section 2.2.2), the MNPs influence on luminescent enzymatic reactions under similar conditions (i.e., in solutions of model oxidizer Bq, *EC*_50_ = 5 × 10^−7^ M) was studied. Similar to bacteria, the effects on bioluminescence intensity were explored in the presence of three types of MNPs–bare Fe_3_O_4_, Fe_3_O_4_/HA, and Fe_3_O_4_/APTES. We did not find any reliable deviations in *I^rel^ _Bq_* (*p* < 0.05) from the control samples (without MNPs) (Figure S5).

The ROS content was measured preliminarily in enzyme-free and enzymatic media, both, under model oxidative stress conditions, i.e., at *EC*_50_ of Bq (5 × 10^−7^ M). It has been shown that Bq increases the content of ROS in enzyme-free and enzyme solutions by 110% and 80%, respectively. Such an increase is probably associated with the formation of intermediate peroxide with the occurrence of bacterial luciferase bioluminescence reaction [117], which contributes additionally to overall ROS content. Dark processes in the reaction of bacterial luciferase with hydrogen peroxide production can contribute to the ROS concentration, as well. The ROS content was monitored in the same media after bioluminescence measurements in the presence of Bq (5 × 10^−7^ M) and MNPs recording unchanged ROS content in enzyme solutions’ overall concentration ranges of MNPs (see Figure S6); no changes were found.

Hence, model oxidative stress has not revealed any reliable changes due to MNPs either in bioluminescence enzymatic intensity or in ROS content in the enzyme solutions. As for the case of absent oxidative conditions (Section 2.3.1), the enzymatic system appeared insensitive to MNP content under oxidative stress conditions, too.

Apparently, all the differences in sensitivity of the bacterial cells and their enzymatic reactions to MNPs are concerned with the cell-membrane processes of luminous bacteria. This result is in agreement with the supposed mechanism of the ferroptosis phenomenon [18,19], which considers the iron-initiated cell-membrane lipid peroxidation in the cellular systems.

## 3. Materials and Methods

### 3.1. Preparations of Fe_3_O_4_ MNPs and Humic Acids- and Amino-Silica Functionalized Fe_3_O_4_ MNPs

Full details regarding the preparation of the samples were previously described [80]. Briefly, magnetite was obtained in accordance with the Elmore reaction. So, FeCl_3_ × 6H_2_O salts (Sigma-Aldrich Chemie GmbH, Steinheim, Germany) and FeCl_2_ × 4H_2_O (Sigma-Aldrich Chemie GmbH, Steinheim, Germany) were dissolved in deionized H_2_O and added to 25% solution of ammonium hydroxide (Sigma-Aldrich Chemie GmbH, Steinheim, Germany) with stirring in argon at 50 °C. The formed Fe_3_O_4_ nanoparticles were washed five times with Millipore water and ethanol and dried at 70 °C in a vacuum.

To prepare the aminosilica-functionalized MNPs, the Fe_3_O_4_ surface modification was performed by using 3-aminopropyltriethoxysilane (APTES, (Sigma-Aldrich Chemie GmbH, Steinheim, Germany)). Fe_3_O_4_ was dispersed in ethanol/water (volume ratio, 1:1) solution, and APTES was added under an argon atmosphere, at 40 °C for 2 h with a molar ratio of APTES:Fe_3_O_4_ as 4:1. After cooling down to a room temperature, Fe_3_O_4_/APTES MNPs were collected with a magnet (Nd, 0.3 T), rinsed three times with ethanol and deionized water and dried in vacuum at 70 °C for 2 h.

Commercial sodium salt of humic acids (HA) (Powhumus, the total acidity of the HA was 5.3 mmol/g of acidic COOH and OH-groups, weight-average molecular weight Mw was 9.9 kD; Humintech, Grevenbroich, Germany) was used for Fe_3_O_4_/HA. For the synthesis of NPs, FeCl_3_ × 6H_2_O and FeCl_2_ × 4H_2_O were dissolved in 100 mL water. Upon heating at 40 °C, two solutions, namely the ammonium hydroxide (25%), and the Has were added rapidly and sequentially. The mixture was stirred at 1000 rpm at 40 °C for 10 min under an argon atmosphere and then cooled to room temperature. The black precipitation of Fe_3_O_4_/HA NPs was collected by Nd-magnet (0.3 T) and washed to neutral by distilled water (90 °C) before drying under a vacuum at 40 °C.

### 3.2. Characterization of the MNPs

Mössbauer spectra were obtained on an MS1104EM spectrometer (CJSC Kordon, Rostov-on-Don, Russia) with ^57^Co/Rh (RITVERC JSC, St. Petersburg, Russia) with an activity of 5 mCi as a source of γ-radiation. The spectra were recorded for each sample both at room temperature (296 K) and at the boiling point of liquid nitrogen (78 K) in an evacuated cryostat. The temperature control accuracy of the samples was ±2 and ±0.5 °C, respectively. The spectra were obtained in high resolution (1024 points) with a noise/signal ratio lower than 2%. Experimental data were processed using SpectrRelax 2.8 and associated software (MSU, Moscow, Russia). The values of chemical shifts in the manuscript are given relative to α-Fe. The magnetic properties of MNP dry powders were haracterized with a Vibrating Sample Magnetometer Lake Shore (Lake Shore Cryotronics, Westerville, OH, USA) at 300 K. To measure the Fe^2+^/Fe^3+^ stimuli-responsive release the obtained samples were dispersed in buffer solutions (dispersed water or 3% NaCl to simulate conditions of bioluminescence assay) containing Potassium thiocyanate (50% solution) and HCl (18.25% solution) as an indicator of Fe^3+^ ions and o-phenanthroline (1% solution) as an indicator of Fe^2+^ ions. At different time points (i.e., 0, 0.5, 1, 3, and 24 h), the mixtures were centrifuged to collect the supernatant. The absorbance in the regions of 490 nm or 690 nm was detected by UV–Vis-NIR spectrophotometry and then analyzed for Fe^3+^ and Fe^2+^, respectively. Elemental analysis of the samples was performed using a CHNS/O elemental analyzer Vario Microcube (Elementar GmbH).

### 3.3. Bioluminescence Assay Systems and Luminol Chemiluminescence Assay

#### 3.3.1. Bioluminescence Assay Systems

Effects of MNPs on microbiological and biochemical processes were evaluated using model bioluminescence assay systems, both cellular and enzymatic, i.e., luminous marine bacteria and a system of coupled enzymatic reactions of the marine bacteria, respectively.

##### Bioluminescence Cellular Assay

The bacterial assay, i.e., intact marine luminous bacteria *Photobacterium phosphoreum*, strain 1883 IBSO from the Collection of Luminous Bacteria CCIBSO 863, Institute of Biophysics SB RAS [118] was used.

For the cultivation of *P. phosphoreum* 1883 IBSO, the semisynthetic medium containing: 10 g/L tryptone, 28.5 g/L NaCl, 4.5 g/L MgCl_2_ × 6H_2_O, 0.5 g/L CaCl_2_, 0.5 g/L KCl, 3 g/L yeast extract, and 12.5 g/L agar was used. *P. phosphoreum* was plated on 25 mL of the semisynthetic medium and incubated at 25 °C for 24 h (stationary growth phase corresponding to the maximum bioluminescence) in an incubator (WIS-20R, WiseCube Laboratory Instruments, Wertheim, Germany). Prior to experiments, bacteria were collected by pipetting of 3% NaCl solution directly onto the agar to release bacteria. The 3% NaCl solution was used to imitate the marine environment for bacterial cells and to balance osmotic processes. The bacterial suspension was diluted to Abs_660_ = 0.025 and stored at 4°C for 30 min to allow the bioluminescence stabilization. The reagents for bacterial cultivation were: tryptone and yeast extract from Dia-M, Moscow, Russia; sodium chloride (NaCl) from Khimreactiv, Nizhny Novgorod, Russia; magnesium chloride hexahydrate (MgCl_2_ 6H_2_O), calcium chloride (CaCl_2_), and potassium chloride (KCl) from Pancreac AppliChem GmbH, Darmstadt, Germany; agar from Difco Laboratories, Detroit, MI, USA. The reagents were of chemical or analytical grade.

Sodium chloride (NaCl) from Khimreactiv, Nizhny Novgorod, Russia was used to prepare a 3% NaCl solution.

##### Bioluminescence Enzymatic Assay

The enzymatic assay, i.e., enzymatic preparation was based on the system of coupled enzyme reactions catalyzed by *NADH:FMN*-oxidoreductase from *Vibrio fischeri* (0.15 a.u.) and luciferase from *Photobacterium leiognathi* at 0.5 mg/mL [118]. The enzyme preparation was produced at the Institute of Biophysics SB RAS, Krasnoyarsk, Russia. Enzyme reactions (1) and (2) are presented in the Introduction.

The used chemicals were FMN and tetradecanal from SERVA, Heidelberg, Germany; NADH from ICN Biochemicals, Costa-Mesa, CA, USA; all reagents were of chemical or analytical grade.

In order to construct the enzymatic assay system, we used 0.1 mg/mL of enzyme preparation, 4 × 10^−4^ M NADH, 5.4 × 10^−4^ M FMN, and 0.0025% tetradecanal solutions. The NADH, FMN, and tetradecanal were dissolved in distilled water, at 25 °C. The concentration of NADH, FMN, and tetradecanal solutions in the experimental samples were 1.6 × 10^−4^ M, 5.4 × 10^−5^ M, and 0.00025%, respectively.

#### 3.3.2. Experimental Data Processing

1. The toxic (for bacterial assay) and inhibitory (for enzymatic assay) effects of MNPs on the bioluminescent systems were characterized by the relative bioluminescence intensity, *I^rel^*:

*I^rel^ = I_MNP_/I_contr_*, (2)

where *I_contr_* and *I_MNP_* are the maximum bioluminescence intensities in the absence and presence of MNPs, respectively.

Values of *I^rel^* were determined at different concentrations of MNPs. The dependence of *I^rel^* vs. MNP concentrations was studied and plotted.

Additionally, to characterize the toxic effects of MNPs on the bacteria, their effective concentrations that inhibited the luminescence intensity by 50% (*I^rel^* = 0.5), *EC*_50_, were determined and compared.

2. The anti-(pro-)oxidant activity of MNPs on the bacterial and enzyme systems was assessed under the conditions of oxidative stress. The methods for determining the anti-(pro-)oxidant activity were elaborated on and developed previously by different authors [67,71,73,75,77,80].

To create conditions of the model oxidative stress, we used an organic oxidizer, 1,4-benzoquinone (Bq) (Sigma-Aldrich, St. Louis, MO, USA). The Bq was prepared in 3% NaCl solutions and in distilled water for bacterial and enzymatic assays, respectively. The Bq concentrations that inhibited the bioluminescence intensity of bacterial and enzymatic systems by 50% were applied in the experiments (10^−7^ M and 5 × 10^−7^ M, respectively). To study and compare changes in the toxicity in the Bq solutions with the addition of MNPs, the antioxidant coefficients *I^rel^_Bq_* were determined as follows:

*I^rel^_Bq_ = I_MNP_ + _Bq_/I_Bq_*, (3)

where, *I_MNP_ + _Bq_* and *I_Bq_* are the maximum bioluminescence intensities in the Bq solutions in the presence and absence of MNPs, respectively. 

A higher MNP concentration range inhibiting the bioluminescence intensity (*I^rel^* < 1) was not used in the experiments to determine *I^rel^_Bq_*. The dependence of *I_MNP_ + _Bq_* vs. MNP concentrations was studied and plotted.

− Values of *I^rel^_Bq_* > 1 revealed a decrease in the toxicity under the exposure to MNPs, i.e., the antioxidant activity of MNPs in the Bq solutions.
− Values of *I^rel^_Bq_* ≈ 1 revealed the absence of the MNP effects.
− Values of *I^rel^_Bq_* < 1 revealed an increase in toxicity under exposure to MNPs, i.e., the pro-oxidant activity of MNPs in the Bq solutions.

#### 3.3.3. Luminol Chemiluminescence Assay

We used the luminol chemiluminescence method to evaluate the content of reactive oxygen species (ROS) in the experimental bacterial suspensions, enzymatic solutions, and non-biological systems [119,120]. This technique was used to determine the integral content of ROS assuming that there was a dynamic equilibrium between different ROS forms.

The reagents used for the chemiluminescence measurements were the following: luminol (C_8_H_7_N_3_O_2_) and potassium ferricyanide (K_3_[Fe(CN)_6_]) from Sigma-Aldrich (St. Louis, MO, USA), potassium hydroxide (KOH) from Khimreactiv (Nizhny Novgorod, Russia). All reagents were of chemical grade.

Stock luminol solution (10^−2^ M) was prepared as follows: luminol powder was dissolved in 5 mL in a 1N solution of KOH and then, 5 mL of distilled water was added. The chemiluminescence luminol reaction was initiated by an alkaline luminol solution; the maximum value of chemiluminescence intensity was determined. The concentrations of the alkaline luminol solution and aquatic solutions of K_3_[Fe(CN)_6_] in the experimental samples were 10^−4^ M and 10^−3^ M, respectively. Initially, the dependences of the chemiluminescence intensity on the concentration of H_2_O_2_ (Tula Pharmaceutical Factory, Tula, Russia), were determined in distilled water and 3% NaCl solution for the enzymatic and bacterial luminescence systems, respectively; they were used as calibration dependences to evaluate the ROS content in all the experimental samples.

To verify the role of ROS in the biological effects of MNPs, chemiluminescence intensities were measured in the bioluminescence assay systems (bacterial and enzymatic), as well as in bacteria-free/enzyme-free aqueous solutions. The relative values of ROS content (*ROS^rel^*) were calculated as ratios of the ROS content in the experimental solutions to that in the control solutions without MNPs:

*ROS^rel^ = ROS_MNP_/ROS_contr_* (4)

The *ROS^rel^* values were obtained at different concentrations of MNPs (10^−15^–1 g/L) in the absence and presence of Bq at *EC*_50_ (see Section 3.3.3). The chemiluminescence assay was carried out immediately after the bioluminescence measurements in the bacterial and enzymatic samples.

The values of *ROS^rel^* were plotted vs. the concentration of MNPs.

#### 3.3.4. Preparation of the MNP Suspensions for the Bioluminescence Analyses

Solid samples were ground in a mortar and dissolved in a 3% aqueous NaCl solution (for bacterial assay or non-biological systems) or distilled water (for enzymatic assays or non-biological systems). To obtain homogeneous suspensions, the stock suspensions were exposed to ultrasound for 10 min. The MNP solutions of different concentrations (10^−15^–10 g/L) were prepared from the stock suspensions, with the additional 5 s ultrasonic treatment at each dissolution stage. Before the measurements, the dispersions were additionally treated ultrasonically for 5–10 s.

To exclude the effect of the optic filter in the bio-(chemi-)luminescence measurements, the suspensions with optical density higher than 0.1 (at 490 nm and 425 nm, respectively) were excluded. Hence, the effect of the “optic filter” [104] did not skew the results of the bio-(chemi-)luminescence measurements. 

#### 3.3.5. Equipment

Measurements of the bio-(chemi-)luminescence intensities were carried out with a bioluminometer Luminoskan Ascent (Thermo Electron Corporation, Solon, OH, USA) equipped with an injector system. All the luminescence measurements were taken at 25 °C and without pre-incubation.

Absorption spectra of the bacterial and MNP suspensions were recorded using a UVIKON 943 Double Beam UV/VIS Spectrophotometer (Kontron Instruments, Milan, Italy).

Ultrasonic treatment was provided using an Elmasonic EASY 10 bath (Elma Schmidbauer GmbH, Singen, Baden-Wurttemberg, Germany).

#### 3.3.6. Statistical Processing

All bio-(chemi-)luminescence measurements were conducted in five replicates for all the control and MNP solutions. 

The SD-values for *I^rel^*, *I^rel^_Bq_*, *ROS^rel^*, *ROS^rel^_Bq_* were calculated using GraphPad Prism 8 (GraphPad Software, San Diego, CA, USA). They did not exceed 21%, 19%, 20%, and 20%, respectively.

To reveal correlations between the bioluminescence intensity and ROS content, the statistical dependence between the rankings of two variables was analyzed [121], and the correlation coefficients *r* were calculated.

Statistical processing of the results of bioluminescence and chemiluminescence assays was carried out; *p*-values were calculated with GraphPad Prism 8 using ANOVA. The *p*-values were assessed by the Kruskal–Wallis test of two independent sample distributions.

## 4. Conclusions

In the current paper, we used bioluminescence bioassays, cellular and enzymatic, to evaluate the bioeffects of three types of magnetite nanoparticles (MNPs), namely, the pristine bare Fe_3_O_4_, humic acid, and silica (3-aminopropyltriethoxysilane) modified nanoaparticles. Additionally, the toxic and anti-(pro-)oxidant bioeffects of MNPs were analyzed assessing the role of ROS. Bacterial bioluminescence was applied as a signal physiological parameter and the ROS content was evaluated by chemiluminescence luminol assay. Higher toxicity related to bare NMPs, Fe_3_O_4,_ was revealed, with a minimum effective concentration of *EC*_50_ at 0.05 g/L. The inhibition ability of modified nanoparticles at higher concentration intervals (i.e., 10^−2^–1 g/L) was associated with ROS accumulation in the bacteria suspensions, but the effect of bare Fe_3_O_4_ appeared to be more complex and it should be further investigated. Wide MNP concentration ranges (10^−15^–1 g/L) have revealed a higher ROS content in bacterial suspensions compared to bacteria-free media, and this supports the conclusion that ROS production is enhanced by bacteria in the presence of MNPs. Under model oxidative stress conditions (i.e., in the presence of model organic oxidizer 1,4-benzoquinone) and higher concentrations of MNPs (>5 × 10^−5^ g/L), the bacterial bioassay has suggested a pro-oxidant activity of all three types of MNPs, with a corresponding decay of ROS content. The ROS decay can be due to shifting the redox balance in the system, such as the reducer-oxidizer model (i.e., 1,4-hydroquinone–1,4-benzoquinone). Bioluminescence enzymatic assay did not show sensitivity to MNPs, and ROS content was unchanged at all applied concentrations of MNPs (<10^−1^ g/L), involving model oxidative stress conditions. The result probably indicates that cell-membrane processes are responsible for the MNP bioeffects and ROS generation by the bacteria, according to the supposed mechanism of ferroptosis phenomenon, which considers the iron-initiated cell-membrane lipid peroxidation in connection of ROS production within the cells.

## Figures and Tables

**Table 1:** Properties of materials according to Mössbauer spectroscopy and Magnetic properties of bare and modified MNPs.

Sample	Composition Fe_3−δ_O_4_ *	D, nm	Saturation Magnetization Ms, emu/g	Remanent Magnetization Mr, emu/g	Coercive Force Hc, Oe
Fe_3_O_4_	Fe_2.718_O_4_	15.64 ± 0.03	68.2	6.88	74.1
Fe_3_O_4_-APTES	Fe_2.734_O_4_	15.73 ± 0.02	31.2	3.93	79.0
Fe_3_O_4_-HA	Fe_2.682_O_4_	12.61 ± 0.06	30.9	6.40	160.0

* δ–calculated as an average from the data in Table S1, D–magnetic domain diameter.

**Table 2:** Effective concentrations of MNPs, *EC*_50_ (i.e., concentrations inhibiting bioluminescence intensity by 50%).

MNPs	*EC*_50_, g/L
Fe_3_O_4_	0.05
Fe_3_O_4_/APTES	0.07
Fe_3_O_4_/HA	0.12

**Table 3:** Correlation coefficients, r, between dependencies of *I^rel^* and *ROS^rel^* on MNP concentrations.

MNPs	MNP Concentration Range, g/L	r
Fe_3_O_4_	(10^−9^–10^−1^)	−0.21
Fe_3_O_4_/APTES	(10^−9^–10^−1^)	−0.88
Fe_3_O_4_/HA	(10^−9^–10^−1^)	−0.79

**Table 4:** Correlation coefficients, r, between dependencies of *I^rel^ _Bq_* and *ROS^rel^ _Bq_* on MNP concentrations.

MNPs	MNP Concentration Range, g/L	r
Fe_3_O_4_	(10^−7^–10^−3^)	0.92
Fe_3_O_4_/APTES	(10^−7^–10^−3^)	0.91
Fe_3_O_4_/HA	(10^−7^–10^−3^)	0.78

## Data Availability

Not applicable.

## Supplementary Materials

The following supporting information can be downloaded at: https://www.mdpi.com/article/10.3390/ijms24021133/s1. References [45,83,122,123,124,125] are cited in the Supplementary Material.

## Abbreviations

APTES	3-Aminopropyltrietoxysilane
Bq	1,4-benzoquinone
FMN	Flavin mononucleotide
HA	Humic acids
MNPs	Magnetite nanoparticles
NADH	Nicotinamide adenine dinucleotide disodium salt-reduced
ROS	Reactive oxygen species
