# Supplementary material for: Functionalized Magnetite Nanoparticles: Characterization, Bioeffects, and Role of Reactive Oxygen Species in Unicellular and Enzymatic Systems
Source: Int J Mol Sci. 2023 Jan 6;24(2):1133. doi: 10.3390/ijms24021133 (PMC9861541; doi:10.3390/ijms24021133)
Supplement: Supplementary file 1 [file ijms-24-01133-s001.zip › ijms-2121374-supplementary.pdf]

SUPPLIMENTARY MATERIALS

Mössbauer spectra at room temperature (296 K) for the Fe_3O_4 and Fe_3O_4-APTES samples have a similar form of distorted sextets (Figure S1), which are asymmetrically broadened due to the manifestation of relaxation phenomena characteristic of small magnetic particles [1]. At the same time, the sextets are asymmetric both in intensity and in width, which indicates the presence of several nonequivalent crystallographic positions of iron atoms in the materials under study. The Mössbauer spectrum at room temperature for the Fe_3O_4/HA sample differs significantly from other samples: its sextet is symmetrical and significantly broadened, so that the intensity of the inner lines exceeds the intensity of the outer ones (Figure S1). These observations indicate that this sample is more uniform in the charge states of iron and is apparently composed of smaller magnetic domains [2]. When the temperature of the samples is lowered to 78 K, the differences between the spectra practically level out – they are asymmetric slightly broadened sextets with an intensity approximately two times greater than at room temperature, which are clearly a superposition of several subspectra.

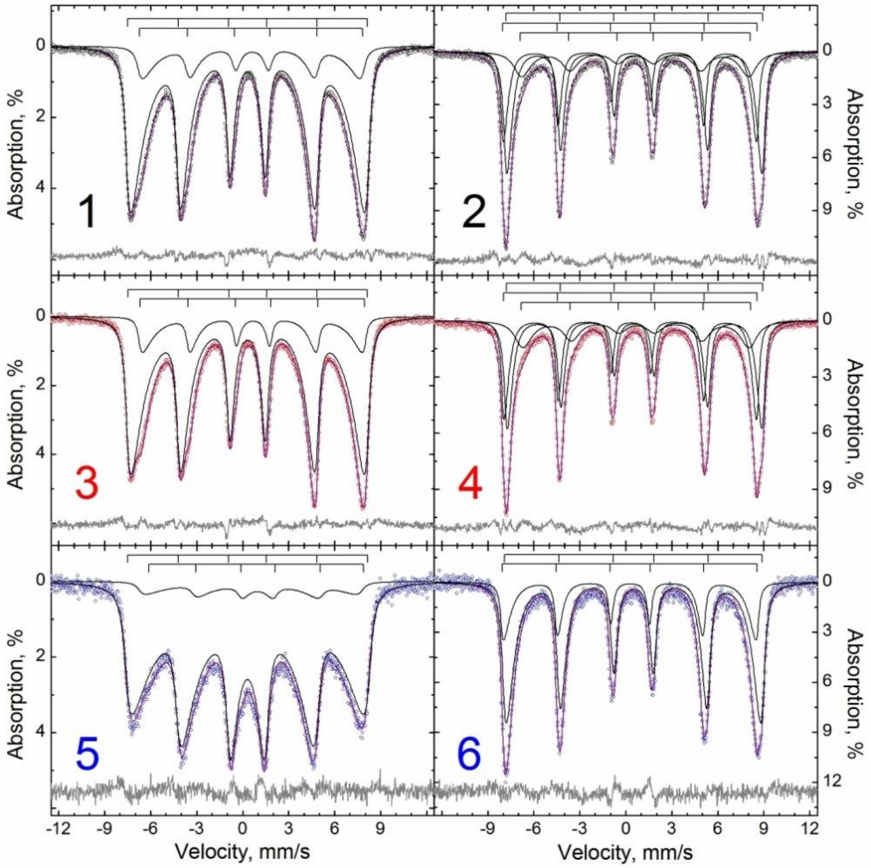

Figure S1. Mössbauer spectra of samples: Fe_3O_4 **(1, 2)**, Fe_3O_4-APTES **(3, 4)** and Fe_3O_4/HA **(5, 6)** at 296 K and 78 K **(1, 3, 5 and 2, 4, 6)**.

Obviously, the reason for these features of the spectra at different temperatures is the manifestation of relaxation phenomena in materials consisting of iron-containing domains of small sizes. This makes it possible to use the many-state superparamagnetic relaxation models to describe the spectra [3] (Figure S1, Table S1). In this case, all samples can be satisfactorily described within the framework of a single model of two at room temperature (296 K) or three (two for Fe_3O_4/HA) at liquid nitrogen temperature (78 K) relaxation sextets related by the ratio of particle anisotropy energy to thermal energy:

$$\alpha=(KV)/(k_BT), \quad \text{(S1)}$$

where K – magnetic anisotropy constant, V – domain volume, k_B – Boltzmann's constant, T – temperature. From equation (S1), the sizes of magnetic domains can be calculated (Table S1), if we assume in the first approximation that they have a spherical shape, and take the value of the magnetic anisotropy constant equal to the “average” value given in the literature for maghemite and magnetite – 10^4 J/m^3 [4,5].

Table S1. Results of the description of the Mössbauer spectra of samples within the framework of the many-state superparamagnetic relaxation model.

Temperature, K		296							78						
Sample	Subspectrum	δ*	E	Γ_{exp}	H_{hf}	S	δ in $Fe_{3-\delta}O_4$	α	δ	ε	Γ_{exp}	H_{hf}	S	δ in $Fe_{3-\delta}O_4$	α
		mm/s			kOe	%			mm/s			kOe	%		
Fe_3O_4	1	0.33±0.01	-0.00±0.01	0.46±0.01	484.9±0.1	83.6±0.6	0.292±0.002	4.92±0.02	0.30±0.01	-0.03±0.01	0.36±0.01	514.2±0.1	31.2±0.7	0.273±0.004	18.60±0.02
	2	0.59±0.01	-0.03±0.01	0.53±0.02	451.8±0.5	16.4±0.6			0.55±0.01	0.01±0.01	0.44±0.01	519.2±0.1	48.5±0.9		
	3								0.62±0.01	-0.01±0.01	1.13±0.02	465.8±0.8	20.3±0.5		
Fe_3O_4/APTES	1	0.33±0.01	-0.00±0.01	0.46±0.01	483.7±0.1	83.4±0.4	0.278±0.003	5.00±0.02	0.32±0.01	-0.02±0.01	0.36±0.01	512.9±0.1	34.6±0.8	0.254±0.005	18.90±0.02
	2	0.66±0.01	-0.02±0.01	0.37±0.01	453.6±0.3	16.6±0.4			0.56±0.01	0.01±0.01	0.43±0.01	518.0±0.1	42.9±0.9		
	3								0.68±0.01	-0.02±0.01	1.15±0.02	464.0±0.7	22.5±0.5		
Fe_3O_4/HA	1	0.33±0.01	0.00±0.01	0.59±0.01	485.0±0.2	96.2±0.5	0.308±0.004	2.6±0.1	0.27±0.01	-0.01±0.01	0.32±0.02	515.1±0.6	26±3	0.3277±0.0004	9.8±0.1
	2	0.95±0.03	-0.07±0.03	0.24±0.07	435±2	3.8±0.5			0.51±0.01	-0.00±0.01	0.45±0.01	521.3±0.3	74±3		

*δ – isomeric shift, ε – quadrupole shift, Γ_{exp} – line width, H_{hf} – hyperfine magnetic field, S – relative area, δ in $Fe_{3-\delta}O_4$ – nanomagnetite nonstoichiometric parameter [6], α is the ratio of particle anisotropy energy to thermal energy.

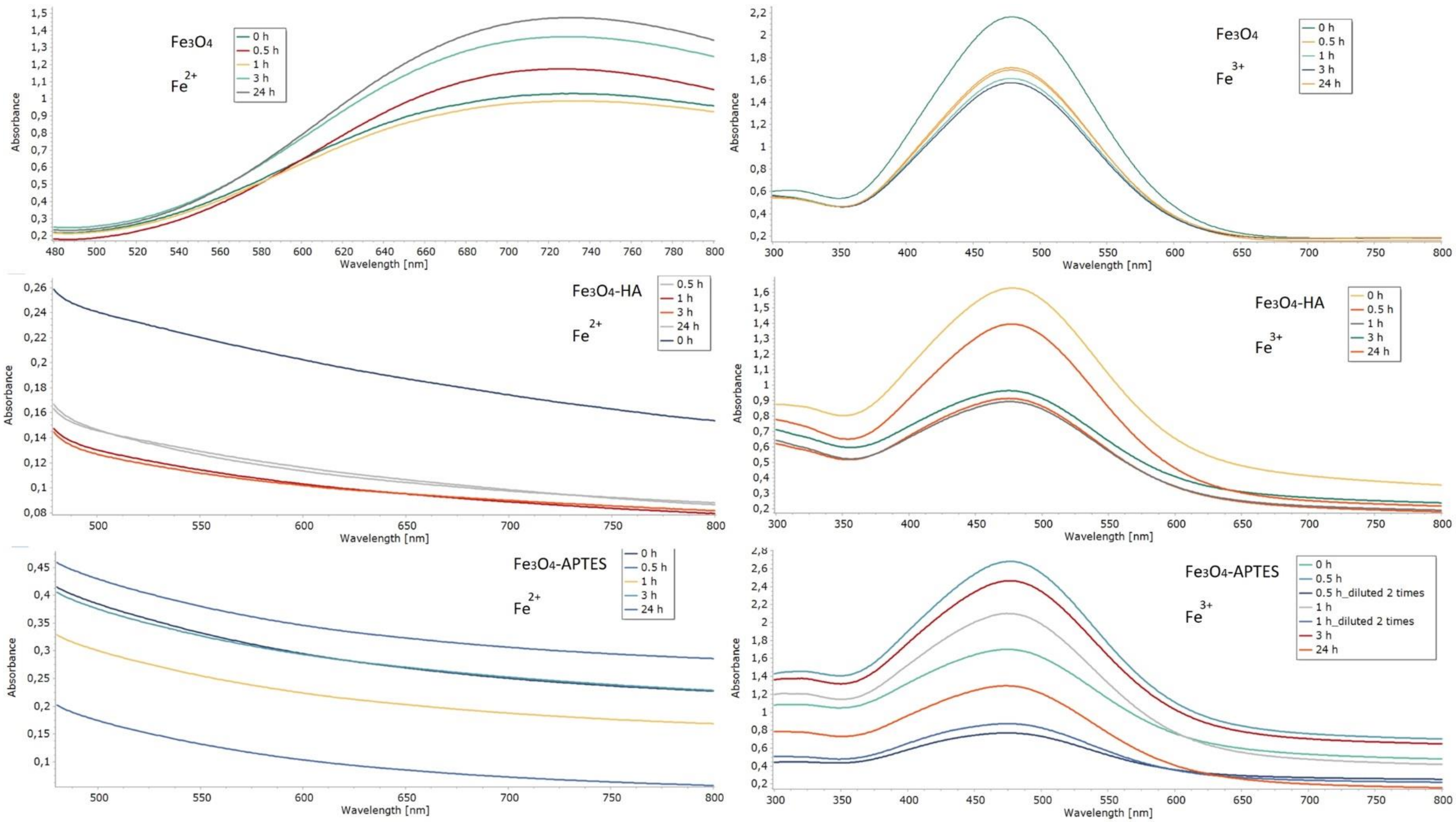

Figure S2. UV-Vis spectra of the release of Fe^{3+} and Fe^{2+} ions by samples (initial sample concentrations: 170 mg/L Fe_3O_4, 43 mg/L Fe_3O_4/APTES and 76 mg/L Fe_3O_4/HA).

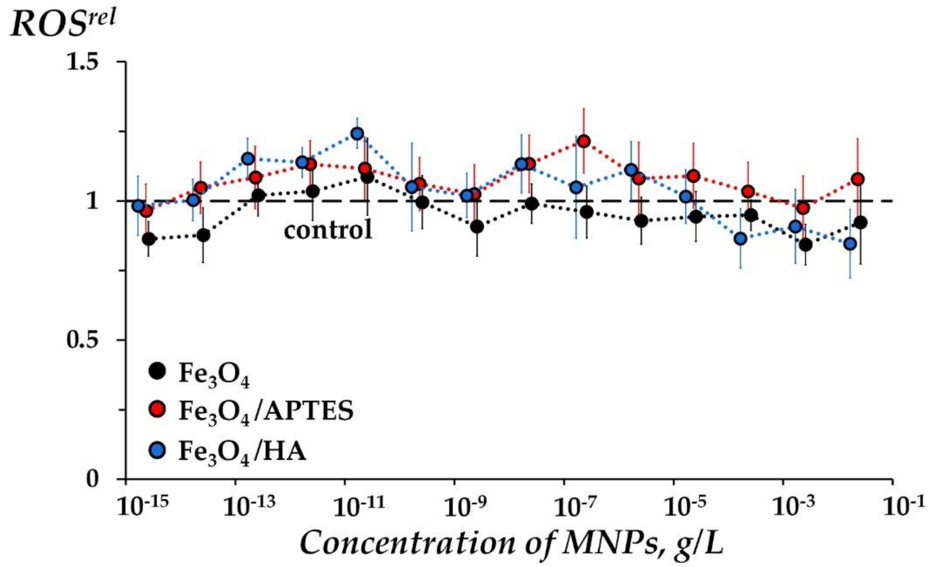

Figure S3. Relative ROS content (*ROS^rel^*) vs. MNP concentration, g/L. Enzyme system. Content of ROS in the control is 1.09×10^{-6} M.

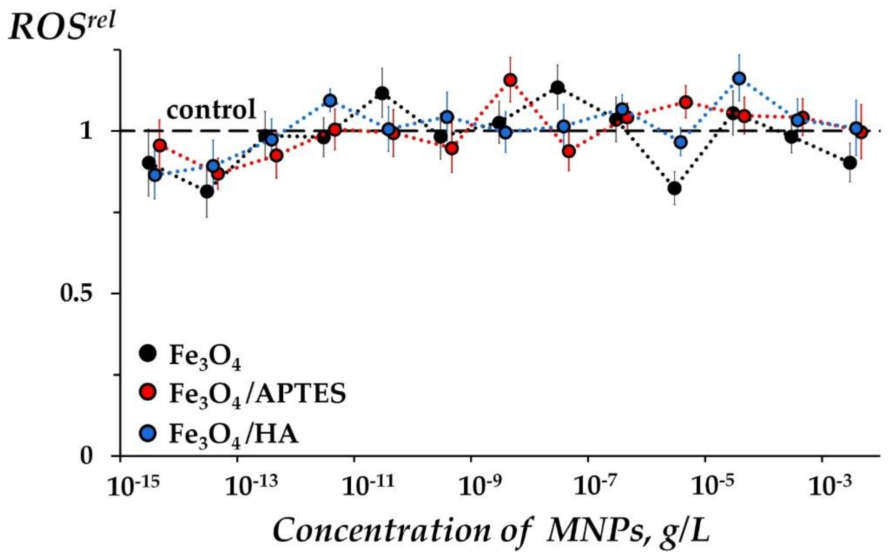

Figure S4. Relative ROS content (*ROS^rel^*) vs. MNP concentration, g/L. Water medium. Content of ROS in the control is $1,95\times10^{-7}$ M).

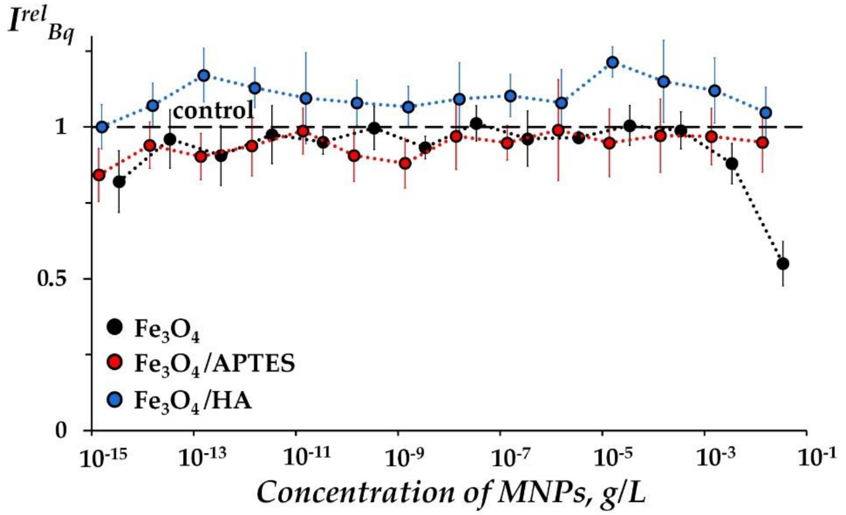

Figure S5. Relative enzymatic bioluminescence intensity, I^{rel}_{Bq}, vs. MNP concentration, g/L, in the presence of Bq ($EC_{50,\ Bq}$ = 5×10^{-7} M).

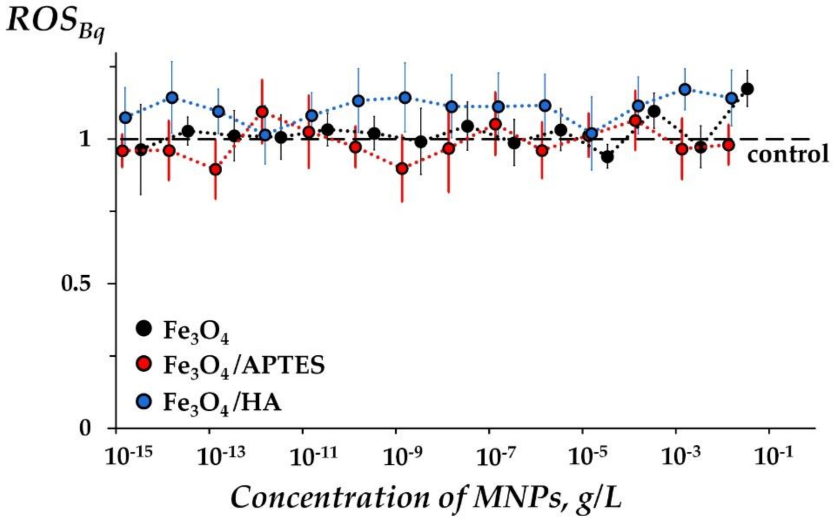

Figure S6. Content of ROS in the enzymatic system at various concentrations of MNPs, g/L, in the presence of Bq ($EC_{50,\ Bq}$ = 5×10^{-7} M). ROS content in the control sample is 5.22×10^{-7} M.

References

1. Rostovshchikova, T.N.; Korobov, M.S.; Pankratov, D.A. Catalytic conversions of chloroolefins over iron oxide nanoparticles 2. Isomerization of dichlorobutenes over iron oxide nanoparticles stabilized on the surface of ultradispersed poly(tetrafluoroethylene). *Russ. Chem. Bull.* **2005**, *54*, 1425–1432, doi: 10.1007/s11172-005-0422-1.
2. Pankratov, D.A.; Anuchina, M.M.; Spiridonov, F.M.; Krivtsov, G.G. $Fe_{3-\delta}O_4$ Nanoparticles Synthesized in the Presence of Natural Polyelectrolytes. *Crystallog. Rep.* **2020**, *65*, 393–397. doi: 10.1134/s1063774520030244.
3. Jones, D.H.; Srivastava, K.K.P. Many-state relaxation model for the Mössbauer spectra of superparamagnets. *Phys. Rev. B* **1998**, *34*, 7542–7548, doi: 10.1103/physrevb.34.7542.
4. Nadeem, K.; Krenn, H.; Traussnig, T.; Würschum, R.; Szabó, D.V.; Letofsky-Papst, I. Effect of dipolar and exchange interactions on magnetic blocking of maghemite nanoparticles. *J. Magn. Magn. Mater.* **2011**, *323*, 1998–2004, doi: 10.1016/j.jmmm.2011.02.04.
5. Adolphi, N.L.; Huber, D.L.; Bryant, H.C.; Monson, T.C.; Fegan, D.L.; Lim, J.; Trujillo, J.E.; Tessier, T.E.; Lovato, D.M.; Butler, K.S.; Provencio, P.P.; Hathaway, H.J.; Majetich, S.A.; Larson, R.S.; Flynn, E.R. Characterization of single-core magnetite nanoparticles for magnetic imaging by SQUID relaxometry. *Phys. Med. Biol.* **2010**, *55*, 5985–6003, doi: 10.1088/0031-9155/55/19/023.
6. Bondarenko, L.S.; Pankratov, D.A.; Dzeranov, A.A.; Dzhardimalieva, G.I.; Streltsova, A.N.; Zarrelli, M.; Kydralieva, K.A. A simple method for quantification of nonstoichiometric magnetite nanoparticles using conventional X-ray diffraction technique. *Mendeleev Communications* **2022**, *32*, 642-644, doi: 10.1016/j.mencom.2022.09.025.
