# Supplementary material for: Functionalized Magnetite Nanoparticles: Characterization, Bioeffects, and Role of Reactive Oxygen Species in Unicellular and Enzymatic Systems
Source: Int J Mol Sci. 2023 Jan 6;24(2):1133. doi: 10.3390/ijms24021133 (PMC9861541; doi:10.3390/ijms24021133)
Supplement: Supplementary file 1 [file ijms-24-01133-s001.zip › Table S1.pdf]

Table S1. Results of the description of the Mössbauer spectra of samples within the framework of the many-state superparamagnetic relaxation model.

**Temperature, K**		**296**							**78**						
**Sample**	**Subspectrum**	**δ***	**ε**	**$Γ_{exp}$**	**H_{hf}**	**S**	**δ in $Fe_{3-δ}O_4$**	**$α$**	**δ**	**ε**	**$Γ_{exp}$**	**H_{hf}**	**S**	**δ in $Fe_{3-δ}O_4$**	**$α$**
			**mm/s**		**kOe**	**%**				**mm/s**		**kOe**	**%**		
Fe_3O_4	1	0.33±0.01	-0.00±0.01	0.46±0.01	484.9±0.1	83.6±0.6	0.292±0.002	4.92±0.02	0.30±0.01	-0.03±0.01	0.36±0.01	514.2±0.1	31.2±0.7	0.273±0.004	18.60±0.02
	2	0.59±0.01	-0.03±0.01	0.53±0.02	451.8±0.5	16.4±0.6			0.55±0.01	0.01±0.01	0.44±0.01	519.2±0.1	48.5±0.9		
	3								0.62±0.01	-0.01±0.01	1.13±0.02	465.8±0.8	20.3±0.5		
Fe_3O_4-APTES	1	0.33±0.01	-0.00±0.01	0.46±0.01	483.7±0.1	83.4±0.4	0.278±0.003	5.00±0.02	0.32±0.01	-0.02±0.01	0.36±0.01	512.9±0.1	34.6±0.8	0.254±0.005	18.90±0.02
	2	0.66±0.01	-0.02±0.01	0.37±0.01	453.6±0.3	16.6±0.4			0.56±0.01	0.01±0.01	0.43±0.01	518.0±0.1	42.9±0.9		
	3								0.68±0.01	-0.02±0.01	1.15±0.02	464.0±0.7	22.5±0.5		
Fe_3O_4-HA	1	0.33±0.01	0.00±0.01	0.59±0.01	485.0±0.2	96.2±0.5	0.308±0.004	2.6±0.1	0.27±0.01	-0.01±0.01	0.32±0.02	515.1±0.6	26±3	0.3277±0.0004	9.8±0.1
	2	0.95±0.03	-0.07±0.03	0.24±0.07	435±2	3.8±0.5			0.51±0.01	-0.00±0.01	0.45±0.01	521.3±0.3	74±3		

*δ – isomeric shift, ε – quadrupole shift, $Γ_{exp}$ – line width, H_{hf} – hyperfine magnetic field, S – relative area, δ in $Fe_{3-δ}O_4$ – nanomagnetite nonstoichiometric parameter [6], $α$ is the ratio of particle anisotropy energy to thermal energy.
