# Supplementary figures and images for: Functionalized Magnetite Nanoparticles: Characterization, Bioeffects, and Role of Reactive Oxygen Species in Unicellular and Enzymatic Systems
Source: Int J Mol Sci. 2023 Jan 6;24(2):1133. doi: 10.3390/ijms24021133 (PMC9861541; doi:10.3390/ijms24021133)

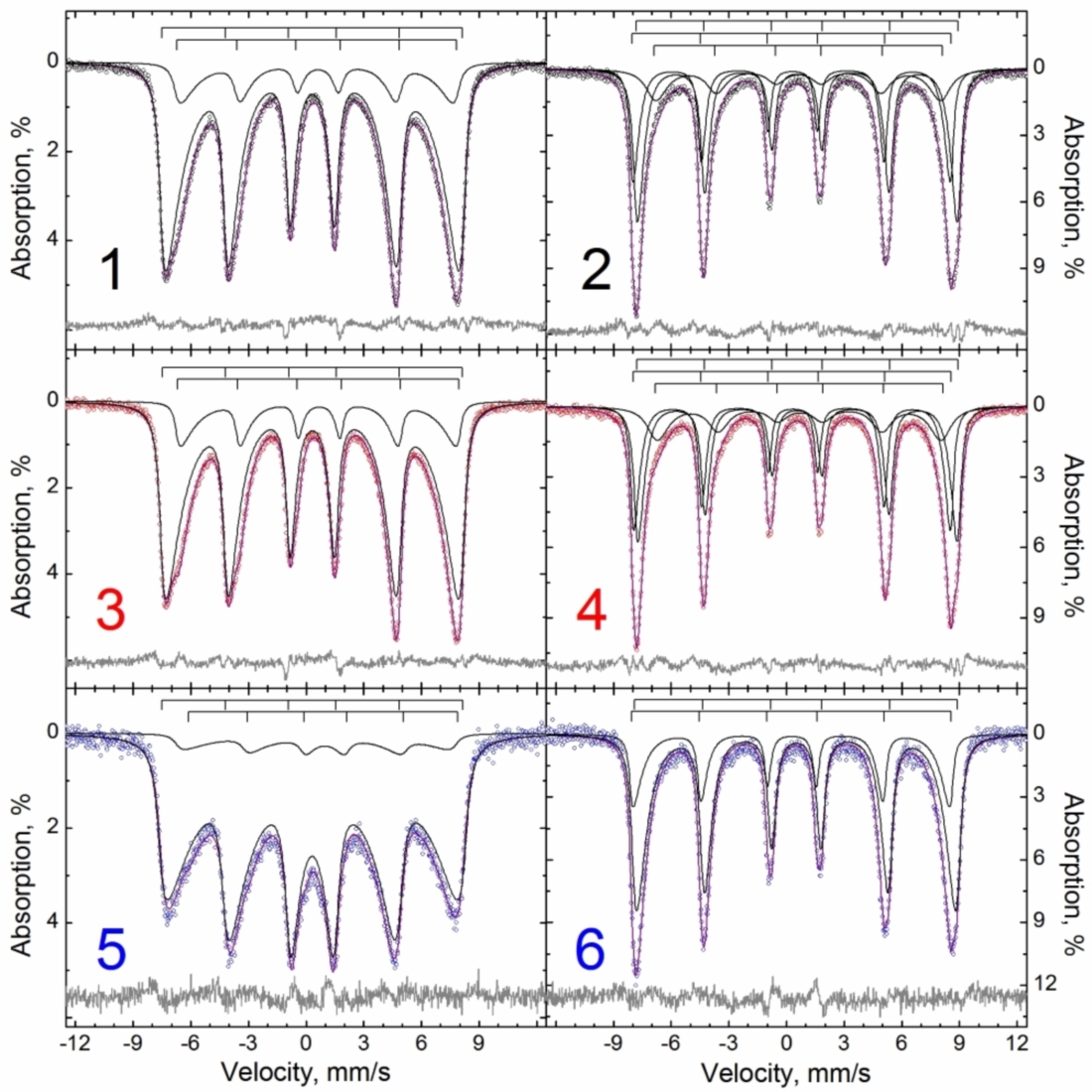

Supplement: Supplementary file 1 [file ijms-24-01133-s001.zip › Figure S1.jpg]

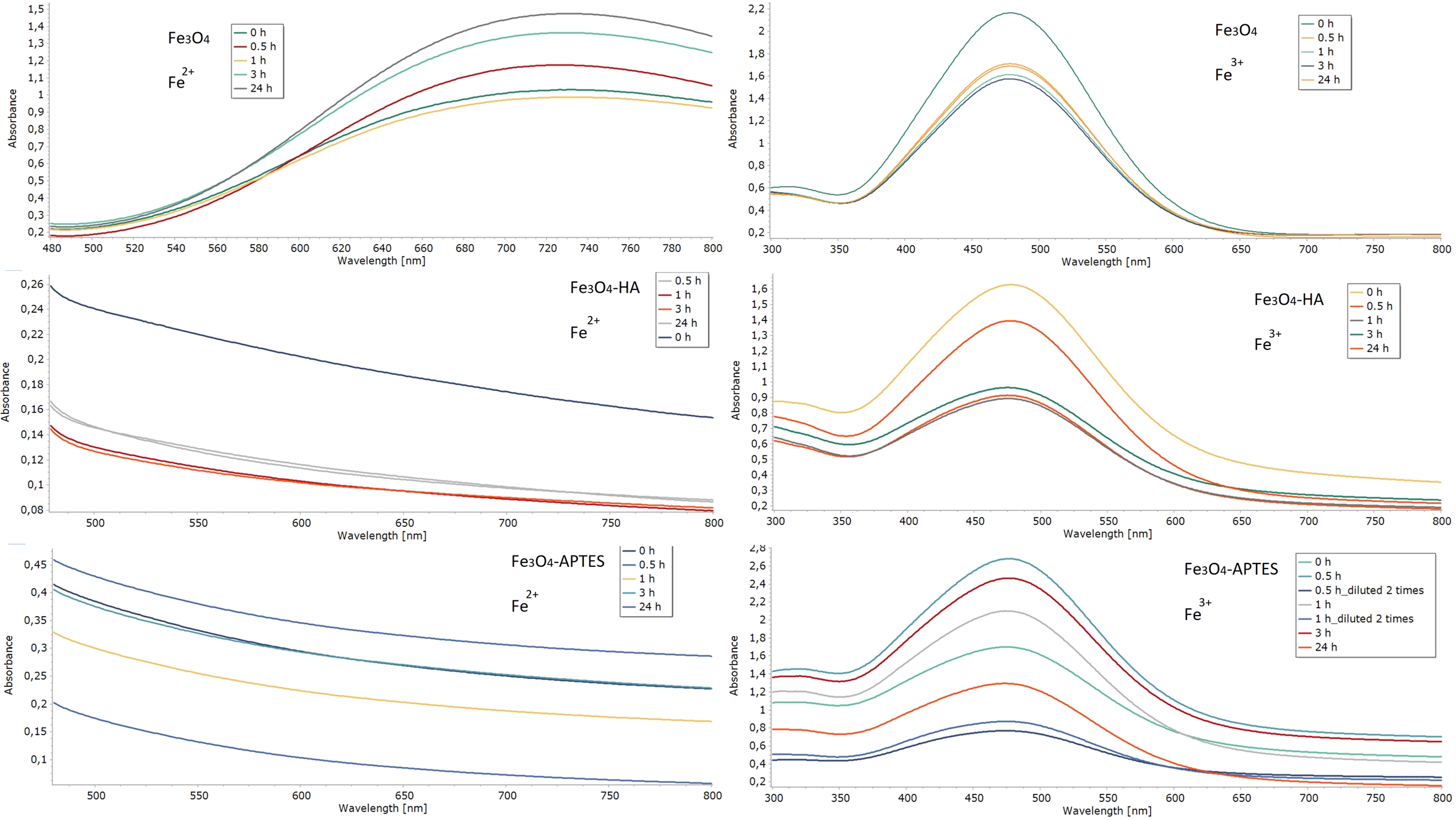

Supplement: Supplementary file 1 [file ijms-24-01133-s001.zip › Figure S2.jpg]

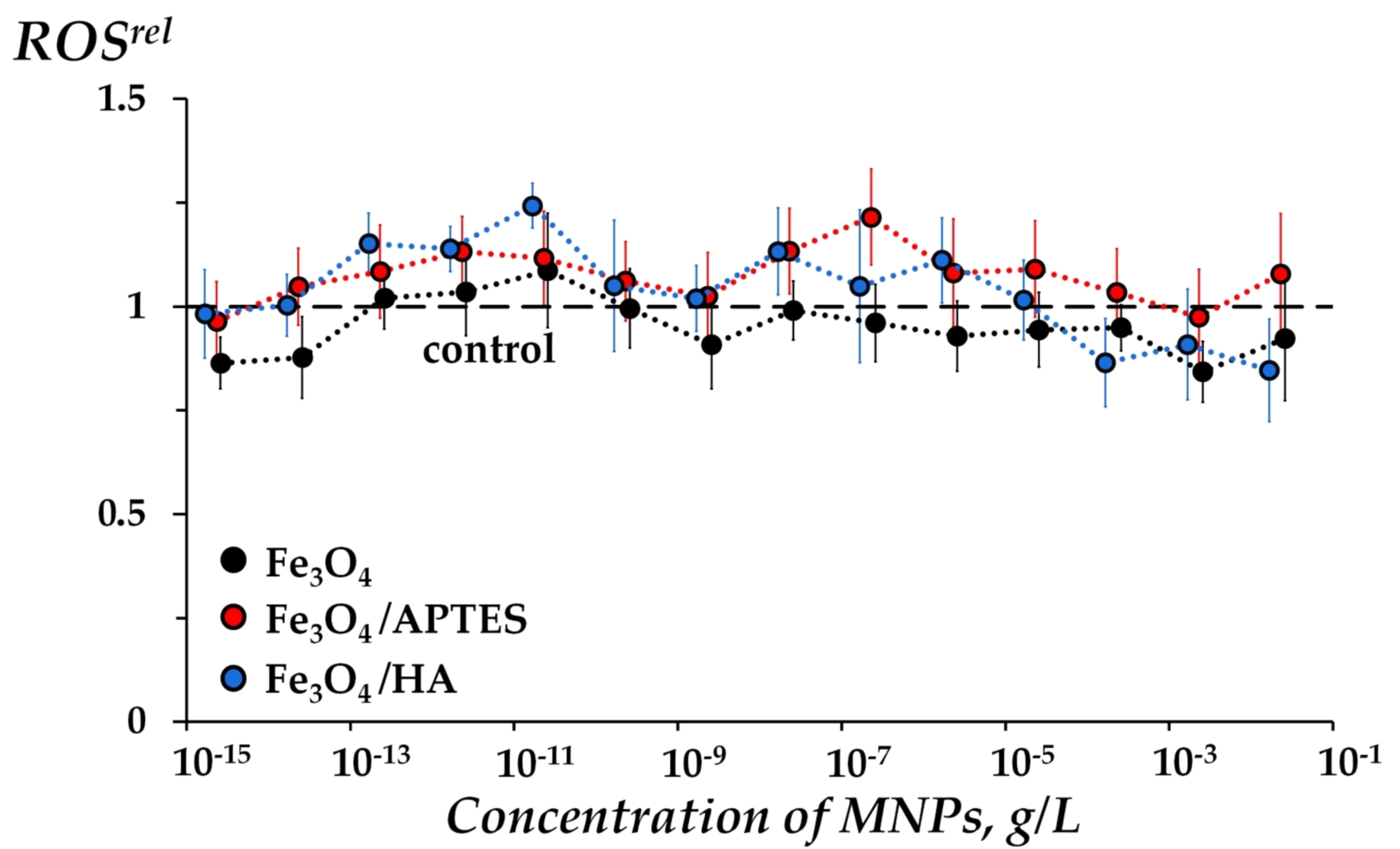

Supplement: Supplementary file 1 [file ijms-24-01133-s001.zip › Figure S3.jpg]

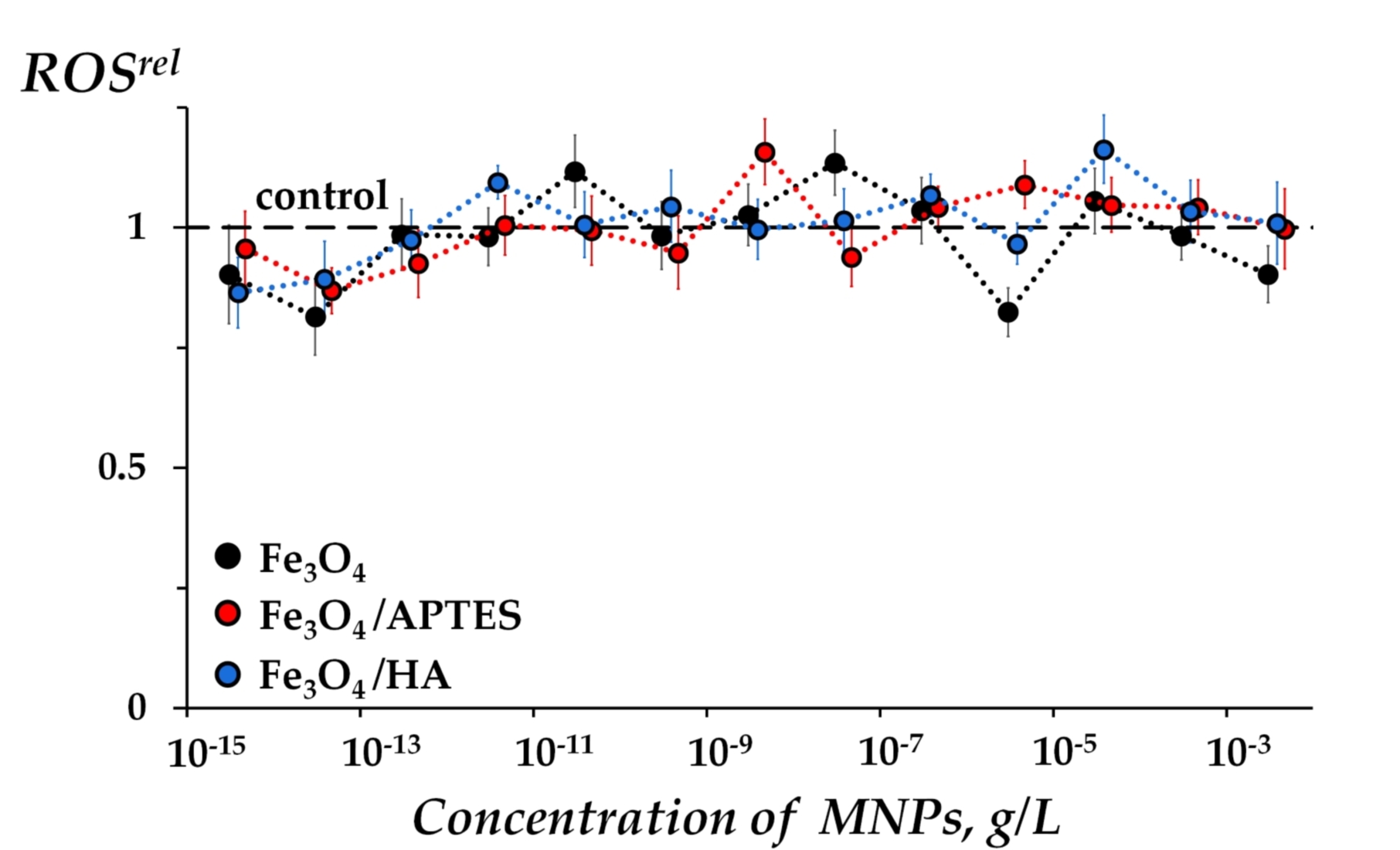

Supplement: Supplementary file 1 [file ijms-24-01133-s001.zip › Figure S4.jpg]

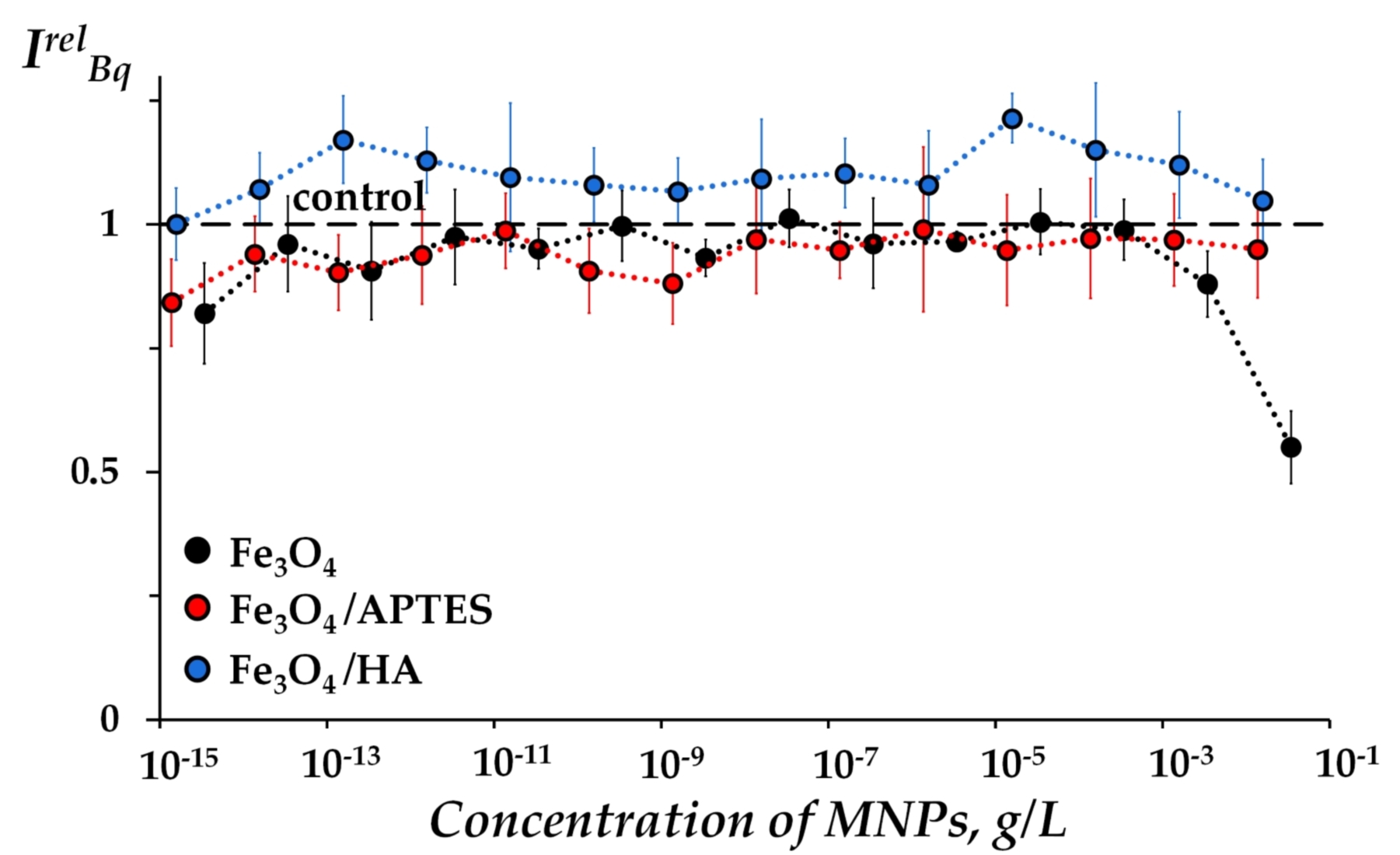

Supplement: Supplementary file 1 [file ijms-24-01133-s001.zip › Figure S5.jpg]

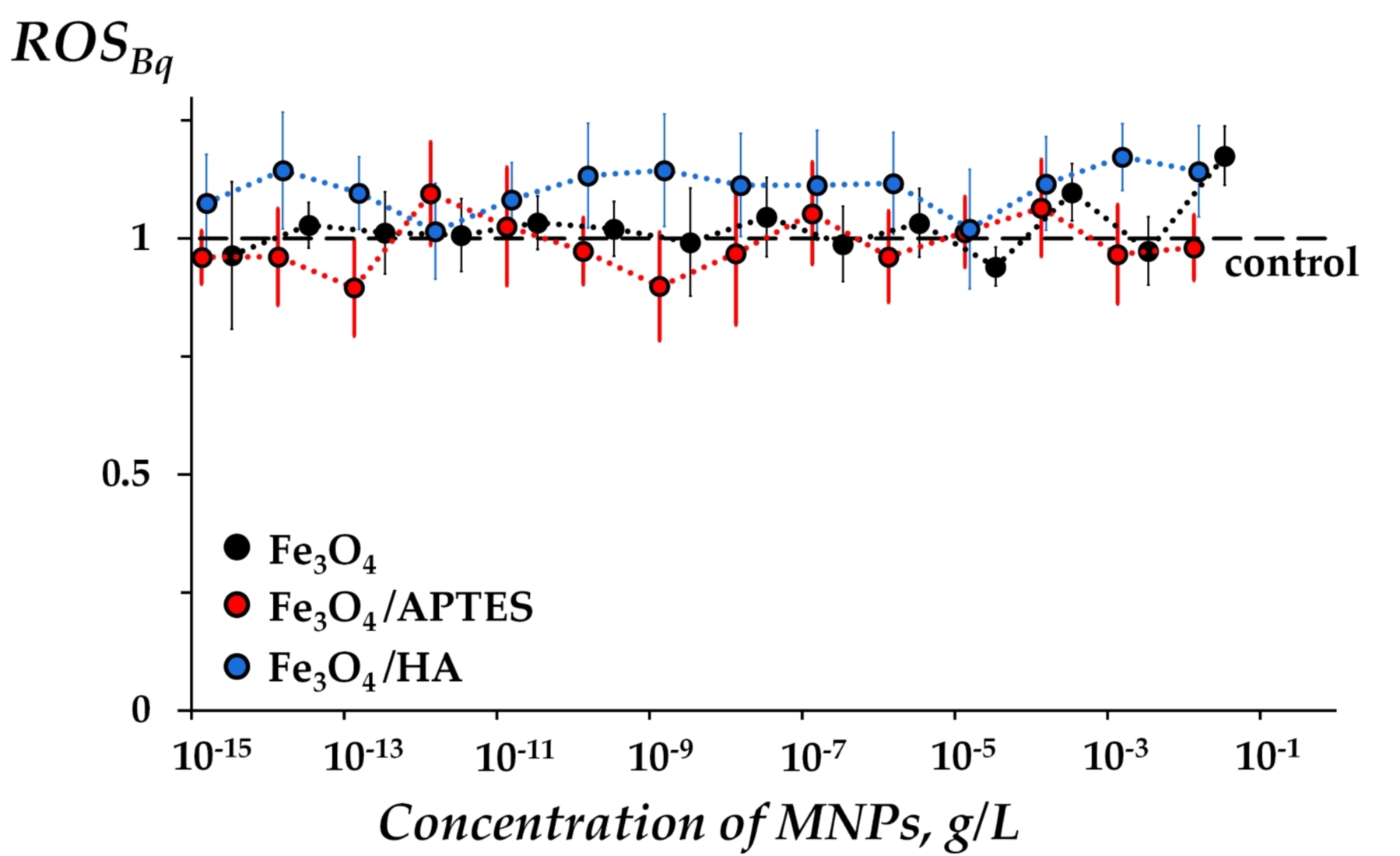

Supplement: Supplementary file 1 [file ijms-24-01133-s001.zip › Figure S6.jpg]
